# Dimerization of melanocortin 4 receptor controls puberty onset and body size polymorphism

**DOI:** 10.3389/fendo.2023.1267590

**Published:** 2023-11-10

**Authors:** Ruiqi Liu, Mike Friedrich, Katherina Hemmen, Kerstin Jansen, Mateus C. Adolfi, Manfred Schartl, Katrin G. Heinze

**Affiliations:** ^1^ Molecular Microscopy, Rudolf Virchow Center for Integrative and Translation Bioimaging, Julius-Maximilians-Universität Würzburg (JMU), Wuerzburg, Germany; ^2^ Developmental Biochemistry, Biocenter, Julius-Maximilians-Universität Würzburg (JMU), Wuerzburg, Germany; ^3^ The Xiphophorus Genetic Stock Center, Department of Chemistry and Biochemistry, Texas State University, San Marcos, TX, United States

**Keywords:** fluorescence lifetime imaging microscopy, Förster Resonance Energy Transfer, Mc4r, puberty, *Xiphophorus*

## Abstract

*Xiphophorus* fish exhibit a clear phenotypic polymorphism in puberty onset and reproductive strategies of males. In *X. nigrensis* and *X. multilineatus*, puberty onset is genetically determined and linked to a melanocortin 4 receptor (Mc4r) polymorphism of wild-type and mutant alleles on the sex chromosomes. We hypothesized that Mc4r mutant alleles act on wild-type alleles by a dominant negative effect through receptor dimerization, leading to differential intracellular signaling and effector gene activation. Depending on signaling strength, the onset of puberty either occurs early or is delayed. Here, we show by Förster Resonance Energy Transfer (FRET) that wild-type *Xiphophorus* Mc4r monomers can form homodimers, but also heterodimers with mutant receptors resulting in compromised signaling which explains the reduced Mc4r signaling in large males. Thus, hetero- *vs.* homo- dimerization seems to be the key molecular mechanism for the polymorphism in puberty onset and body size in male fish.

## 1 Introduction

Puberty is a mechanism ensuring that sexual maturity occurs only when males and females are in the appropriate nutritive status for reproduction. It marks the transition of the juvenile stage to adulthood.

Reproduction is a fundamental feature that impacts life history and reproduction-related phenotypes in morphology, physiology and behavior, *e.g.* timing of puberty onset, adult body size, and reproductive strategies. Reproduction-related phenotypes are often species-specific but can also be polymorphic within one species.

Polymorphism in puberty onset and reproductive tactics are long-known in fish of the genus *Xiphophorus* (1, 2), making these species a classical model for studies on the genetic control of reproduction-related phenotypes. In some species (the swordtails), males develop at puberty an extension of the ventral rays of the caudal fin, which is used in courtship behavior and is a sexually selected trait attractive to females (3, 4). Males of the two best-studied swordtail species, *X. nigrensis* and *X. multilineatus*, display a prominent polymorphism of puberty onset and adult body size (5–8). Natural populations consist of large, intermediate and small males. Since males cease to grow at puberty onset, early-maturing fish are small and late-maturing fish are large in size. Females continue to grow throughout the life.

Age at puberty onset is a critical time point in life history of an individual. It influences the body size, lifetime reproductive success as well as survival rate. Puberty onset age is a classic evolutionary trade-off and the polymorphism is maintained by balancing selection (9). Larger, later-maturing males show elaborate courtship behavior, are territorial, preferred by females and thus have higher reproductive success. However, they also experience a higher risk of being caught by predators before reaching maturity and during courtship display. Smaller, earlier-maturing males compensate the relative lower reproductive success rate by lower survival risks of being prey and sneak mating behavior. In the end, the lifetime reproductive success is balanced between early-maturing small and late-maturing large males (9).

**Figure 1:**
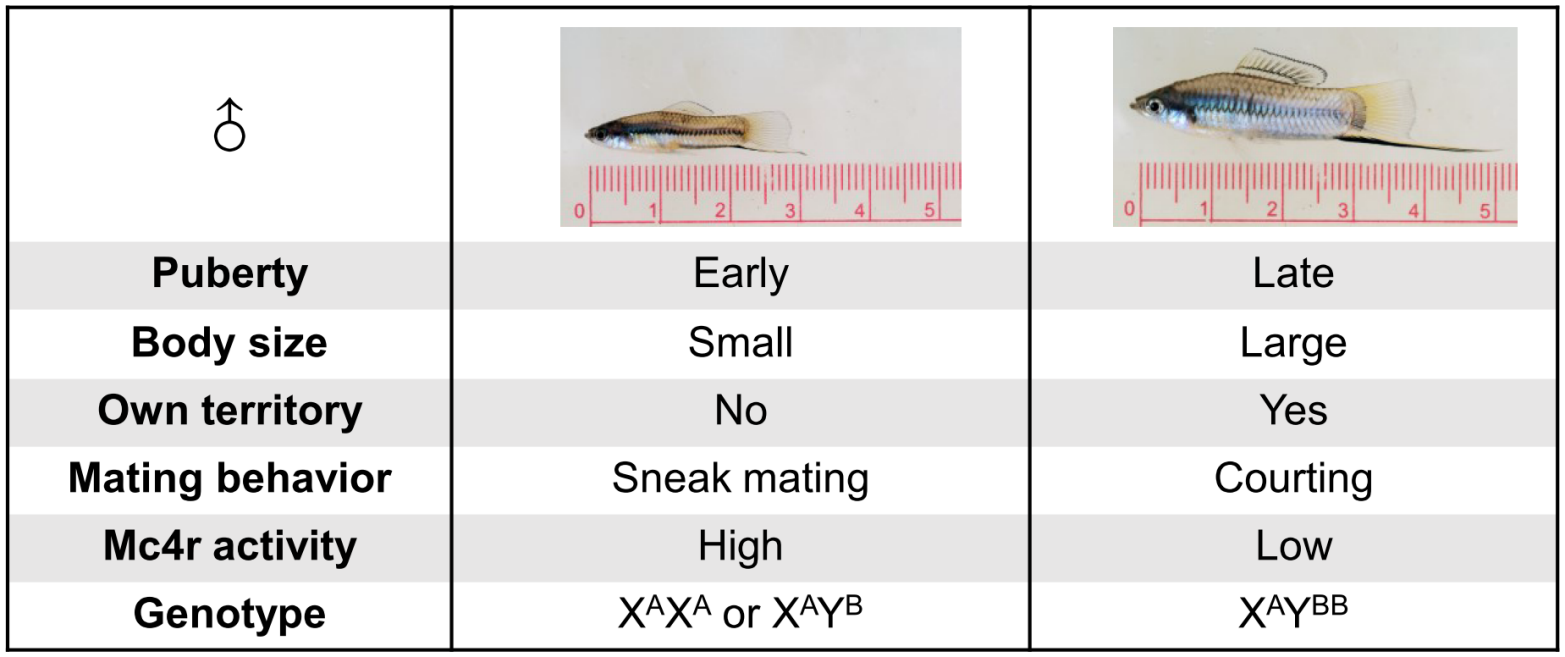
Illustration of key differences between small and large males of *X. nigrensis* and *X. multilineatus*. Genetic polymorphism of *mc4r* controls the polymorphism in fish puberty onset timing, body size and reproduction tactics. Large males have more Y-chromosomal *mc4r* B allele copies (here shown as two B) than small males.

Though both, environmental and genetic factors collectively determine the polymorphism of reproduction; in *Xiphophorus* fish, a single Mendelian locus, called *P* locus (*Puberty* locus), controls puberty onset of males. A previous study identified the melanocortin 4 receptor (*mc4r*) gene as the *P*-locus encoded gene (5). Allelic and copy number variation of *mc4r* at the *P* locus is causative for the reproduction-related polymorphisms (**Figure 1**). Mc4r comprises three alleles, the wild-type A alleles and non-signal-transducing mutant B1 and B2 alleles. The A alleles are present in both males and females and are located on the X chromosome. The B alleles are found only in males and assigned to the Y chromosome. Compared to small males, large males have higher copy number of B alleles and higher *mc4r* expression. Based on *in vitro* assays of co-expressing A and B1 isoforms, B alleles elicit lower cAMP signaling. This has led to the hypothesis that the B isoforms may act on A isoforms by a dominant negative effect (5, 10).

Mc4r belongs to class A G-protein coupled receptors (GPCR) with the typical seven-transmembrane helix structure. Mc4r A and B isoforms differ at the carboxy-terminus (5). B1/B2 lack two cysteine residues, which are considered to anchor the carboxy-terminal helix VIII of the wild-type protein in the inner layer of the plasma membrane. The B2 allele has in addition a frameshift after the cysteine residues, resulting in an elongated carboxyl terminus. Besides the receptor itself, the Mc4r signaling system includes agonists melanocortins, antagonist agouti-related peptide (Agrp), and melanocortin receptor accessory protein 2 (Mrap2) (11). Melanocortins are processed from the precursor pro-opiomelanocortin (Pomc), and include α-MSH, β-MSH, γ-MSH, and ACTH; however, teleosts lack γ-MSH (12). Among them, β-MSH has the highest affinity to Mc4r. The synthetic ligand NDP-α-MSH is a highly potent agonist for Mc4r (13). Agrp acts as both antagonist and inverse agonist, inhibiting the induced activity and constitutive activity, respectively (14). Mrap2 interacts with Mc4r. In *X. nigrensis* and *X. multilineatus* fish, despite Mrap2 increasing constitutive and ligand-induced Mc4r signaling, probably by stabilizing an active conformation of Mc4r, Mrap2 is not mediating differential Mc4r signaling as basis of body size polymorphism (10).

Mc4r plays a central role in energy homeostasis. In human, mutations in *MC4R* are the most common cause of early-onset obesity (15). Over 200 distinct mutations have been identified in various human patients, and the frequency of *MC4R* mutations in cohorts of obese patients is around 5% (16–18) and *MC4R* loss-of-function mutations is about 0.3% in general population from a UK study (19). Receptor dimerization has been reported for human MC4R (20), and *MC4R* mutations which have impaired the dimerization are obesity-associated (21). Thus, dimerization might be a potential mechanism to explain the pathophysiology of the disease in human. Since the nutritional status is key for the onset of puberty, energy homeostasis is a relevant determinant in this process and the known role of Mc4r supports its function as the *P*-locus gene. Yet the molecular basis how B alleles could act as dominant negative versions on A alleles remains to be elucidated. Here, we test the hypothesis that the receptors encoded by the A and B alleles form heterodimers. Such heterodimerization could explain the dominant negative effects of B in diminishing the signaling from A allele encoded isoforms. Moreover, it is unknown whether Mc4r also forms homodimers and whether such a homodimerization influences equilibrium of heterodimers. To test this hypothesis, we used Förster Resonance Energy Transfer (FRET) of fluorescent protein tagged receptors in a cell-based approach. FRET has unique sensitivity in the nm distance range, and thus is widely used to study protein dimerization (22). The fluorescence characteristics change upon dimerization when one fluorophore (donor) transfers the excitation energy to the other fluorophore (acceptor) through nonradiative dipole–dipole interactions. Fluorescence Lifetime Imaging Microscopy (FLIM) based FRET (23) is particularly robust against intensity fluctuations and photobleaching, and thus well-suited for the present study. In addition to the information from FRET efficiency, pulsed interleaved excitation (PIE) (24) based FRET-FLIM can measure donor and acceptor simultaneously and retrieve the information of stoichiometry of the formed receptor complexes. Assessing the stoichiometry allows to estimate the dimerization grade even without knowledge of absolute protein concentrations (25). Here, we detect homodimers (of Mc4r wild-type A or mutant B receptors) and heterodimers (A-B dimerization) as the key to pinpoint the low receptor signaling in the large males.

## 2 Materials and methods

### 2.1 Molecular cloning

The different *mc4r* alleles (A, B1, B2) of *X. multilineatus* were identified and cloned previously (5). To detect the dimerization of receptor monomers by FRET, expression vectors were designed as receptor genes tagged at the C-terminus with eGFP or mCherry as FRET sensors. Fluorescent proteins eGFP and mCherry were cloned into a pcDNA3 vector between restriction sites *Xba*I and *Not*I. The receptor open reading frames (ORFs) were PCR amplified (primers listed in **Supplementary Table 1**), digested with restriction enzymes *Hind*III (R3104S, New England Biolabs, Ipswich, Massachusetts, United States) and *Xba*I (R0145S, New England Biolabs), and ligated to backbones pcDNA3-eGFP/mCherry in frame with eGFP/mCherry encoding sequences. CD28, a dimeric cell-surface receptor was used as a reference dimer (positive control) and β1-adrenergic receptor (β1AR) was used as a reference monomer (negative control) (26). Both CD28 and β1AR were cloned from SNAP-tag constructs (kindly provided by Prof. Martin Lohse) into pcDNA3-eGFP/mCherry backbones in the same way as Mc4r receptors. To compare the fish Mc4r with its human orthologue, human MC4R wild-type and mutant (chim7) were also used as references (27). All receptors were cloned by the above-mentioned procedures, and all recombinant plasmids were verified by sequencing.

### 2.2 Cell culture

Human embryonic kidney 293T (HEK293T) cells were cultured in Dulbecco’s modified Eagle medium (DMEM) without sodium pyruvate (P04-03550, PAN Biotech, Aidenbach, Germany), with 10% fetal calf serum (FCS) (AC-SM-0190, Anprotec, Bruckberg, Germany) and 100 U/mL penicillin, 100 µg/mL streptomycin (P4333, Sigma-Aldrich, St. Louis, Missouri, United States) at 37°C, 5% CO_2_. To passage the cells, culture medium was aspirated; cells were washed with PBS (14190, Gibco, Thermo Fisher Scientific, Waltham, Massachusetts, United States) once, and cells were detached using 0.5× Trypsin-EDTA solution (T4299, Sigma-Aldrich). The cell suspensions were resuspended in DMEM culture medium. Cells were regularly passaged every 3-4 days.

### 2.3 Fluorimetric cAMP assay

To confirm the functionality of fluorescent protein tagged receptors, cAMP response of selected constructs was monitored using cAMP Direct Immunoassays Kit (Fluorometric) (ab138880, Abcam, Cambridge, United Kingdom). HEK293T cells were seeded at 50 000 cells/well in a poly-D-lysine (P6407, Sigma-Aldrich) coated 96-well plate (82.1581.001, Sarstedt Inc., Newton, North Carolina, United States). On the next day, cells were transfected by poly-ethylenimine (PEI) (23966, Polysciences Inc., Warrington, Pennsylvania, United States) with eGFP-tagged receptor constructs for best compatibility of the plate reader. In total 0.5 µg of plasmids were used for six samples, *i.e.* 83.3 ng for each well in the 96-well plate. Controls included original Mc4r-A construct without tags (positive control) and pcDNA3 mock transfection (negative control). Six to eight hours post transfection, cells were washed and activated by agonist NDP-α-MSH (M8764, Sigma-Aldrich) at a final concentration of 100 nM. Cells were incubated with NDP-α-MSH overnight for 15-17 h. On the third day, cells were harvested and cAMP was detected using the cAMP direct immunoassay by CLARIOstar plate reader (BMG Labtech, Ortenberg, Germany), operated by CLARIOstar software, following the manufacturer’s protocols. In short, cells were lysed and collected; cAMP standard and cell lysate samples were loaded on a black 96-well plate coated with anti-cAMP antibodies. HRP-cAMP was added to each standard and sample well, and incubated for 2 h, followed by extensive washing. Detector AbRed, a fluorophore capable to react with HRP-cAMP, was added to each well and incubated. Fluorescence was monitored (exc. 540 ± 8 nm, em. 590 ± 8 nm). The observed fluorescence is proportional to the activity of HRP-cAMP, hence it is negatively correlated to cAMP concentration from the samples. Standard curve was fitted with modified Hill function with offset in OriginPro 2021 (OriginLab Corporation, Northampton, Massachusetts, United States). cAMP concentrations from the samples were determined from the fitted standard curve. All values were calculated from three independent experiments.

The modified Hill equation (n=1) is given by:


(1) $$RFU=RFU_{max}+(RFU_{min}-RFU_{max})\times \frac{c^{n}}{k^{n}+c^{n}}$$

Where RFU is the fluorescent intensity of sample at cAMP concentration, c . $RFU_{max}$ and $RFU_{min}$ are the maximum or minimum fluorescent intensity from the samples of lowest or highest cAMP concentrations, respectively. k stands for cAMP concentration producing at half occupation.

### 2.4 Confocal imaging

To verify correct receptor localization in cells, tagged receptors were transfected into cells and visualized by confocal imaging. HEK293T cells were seeded at 500 000 cells/well on Ø24 mm #1 coverslips (0111640, Marienfeld-Superior, Lauda-Königshofen, Germany) in 6-well plates (140675, Thermo Fisher Scientific, Roskilde, Denmark) and grown until 50-70% confluency. Cells were transiently transfected by PEI reagent to express the eGFP/mCherry tagged receptor constructs. Two days after transfection, cells were fixed by 4% para-formaldehyde (PFA) (158127, Sigma-Aldrich) and mounted in Mowiol 4-88 (0713.1, Roth, Karlsruhe, Germany). Imaging was performed with a laser scanning confocal microscope (TCS-SP5 Leica, Wetzlar, Germany), operated by LAS AF software, equipped with a 40×/1.35 NA oil objective [eGFP and mCherry exc. at 488 nm and 594 nm, respectively; em. detected at 500-550 nm and 600-700 nm, respectively, by a Photomultiplier tube (PMT)]. Images were collected with 400 Hz line scanning, zoom 9.0 and line average of 8. Each image had 512×512 pixel with a pixel size of 0.084 µm.

### 2.5 FRET imaging sample preparation

HEK293T cells were seeded at 500 000 cells/well on poly-D-lysine coated Ø24 mm #1 coverslips in 6-well plates and grown for ~23 h. Cells were transiently transfected by PEI reagent to express eGFP and/or mCherry tagged receptors. Dimerization of receptors was probed by using eGFP as donor fluorophore and mCherry as acceptor fluorophore. Samples for FRET imaging include donor-only samples, acceptor-only samples, and FRET samples with donor/acceptor ratio 1:1 and 1:5 at transfection. The two different transfection ratios can explore a decent donor/acceptor dynamic range. Total DNA amount was kept constant, using 2 µg per well in 6-well plates. After overnight incubation (~18 h), cells were fixed by 4% PFA and mounted in Mowiol 4-88. Three independent experiments were performed.

To probe dimerization in the experiments, the Mc4r A/B1/B2 protomers were combined in main permutations, both homodimers (A-A, B1-B1, B2-B2) and heterodimers (A-B1, A-B2, B1-B2). Here, β1AR served as a monomeric control, and CD28 as a constitutive dimeric control. To understand the equilibrium of monomer *vs.* dimer formation, heterodimer formation concerning A and B1 protomers were probed by A-eGFP/B1-mCherry (A-B1) and B1-eGFP/A-mCherry (B1-A). Depending on the affinity of A-A and B1-B1 homodimers, the equilibrium towards A-B1/B1-A heterodimers in a mixture may differ.

### 2.6 FRET acceptor photobleaching

Fixed cell samples were imaged by laser scanning confocal microscopy (Leica TCS-SP5, see subsection **confocal imaging** above) with the same settings mentioned above. Intensity-based FRET measurements were performed by acceptor photobleaching approaches (FRET-AB) according to manufacturer’s wizard tools (FRET AB Wizard in LAS AF software). In brief, a region of interest (ROI) was defined manually to include an extended membrane region, which mimics basal membrane. Before and after the bleach of ROI, images were acquired in both 488 nm and 594 nm channels. Bleach was done at 594 nm by 60% power for four frames. The intensity of the ROI was recorded and used for calculating intensity-based FRET efficiency.

The intensity-based FRET efficiency $E_{int}$ is given by (28):


(2) $$E_{int}=1-\frac{I_{pre}^{D|D}}{I_{post}^{D|D}}$$

Whereby $I_{pre}^{D|D}$ and $I_{post}^{D|D}$ are the uncorrected signal intensities of donor in the presence of acceptor (before photobleaching) and in the absence of acceptor (after photobleaching), respectively.

### 2.7 FRET fluorescence lifetime imaging microscopy

Time-resolved FRET measurements (FRET-FLIM) were performed on a Zeiss LSM980 confocal microscope (Zeiss, Oberkochen, Germany), operated by ZEN3.2 software, equipped with the LSM upgrade kit (PicoQuant, Berlin, Germany), operated by SymPhoTime64 software. The confocal and the time-resolved fluorescence features were controlled by Zeiss Blue Picoquant Application software. Fixed cell samples were imaged under 63×/1.4 NA oil objective. Pulsed lasers at 480 nm and 560 nm were used for excitation, and detection was achieved by photon counting detectors PMA Hybrid 40 (Picoquant, Berlin, Germany) with bandpass filters at 520/35 nm and Excelitas SPCM-AQRH-14-TR (Picoquant, Berlin, Germany) with bandpass filter at 600/50 nm. Acquisition was performed at a zoom factor of 3.0 and with a pixel size of 0.088 µm; the image size was 512×512 pixel. A pixel dwell time of 0.85 µs was used. For a FLIM measurement, the repetition rate was set to 40 MHz and pulsed interleaved excitation (PIE) (24) was applied at a delay time of 25 ns and a whole time window of 50 ns to alter donor and acceptor excitation. Laser power was set to 60-4100 nW for 480 nm laser and 30-2100 nW for 560 nm laser. Time-correlated single photon counting (TCSPC) resolution was 10 ps. On each experimental day, an Erythrosine B (200964, Sigma-Aldrich) saturated in potassium iodide (KI) (221945, Sigma-Aldrich) solution was used to measure the instrument response function (IRF). Donor-only and FRET samples were measured using basal or apical membrane of the cell for 180 s (with exception of longer measurement when donor/acceptor expression level is much lower). The excitation intensity of the lasers was adjusted such that the donor channel count rate was about 50 kcps (50 000 counts per second), the excitation intensity ratio between the donor and acceptor excitation was kept constant.

### 2.8 Live cells FRET-FLIM with agonist activation

To investigate if receptor activation would change the dimerization, FRET-FLIM on live cells samples were performed. HEK293T cells were seeded at 150 000 cells/well on poly-D-lysine coated 4-chambered coverglasses (C4-1.5P, Cellvis, Mountain View, California, United States) and grown for ~18 h. Cells were transiently transfected by PEI reagent to express either donor-only samples or FRET samples (A-A and A-B1) with donor/acceptor ratio 1:1. Six to eight hours post transfection, cell culture medium was changed and cells were incubated overnight. On the next day, cell medium was changed to imaging medium [DMEM without phenol red (P04-01161, PAN Biotech), 15 mM HEPES (15630-080, Gibco), 10% fetal calf serum, 2 mM glutamine (G7513, Sigma-Aldrich)], which allows for 2-3 h of live cell imaging without CO_2_ source. After mounting the coverglass on the Zeiss LSM980 confocal microscope (see subsection FRET fluorescence lifetime imaging microscopy above), the first FRET-FLIM measurement (preadd) was taken, without the agonist. NDP-α-MSH was then applied into the well to final concentration of 100 nM. Four FRET-FLIM measurements (postadd) were taken from the same cell at 5 min/10 min/15 min/20 min after adding of ligand. The FRET-FLIM measurements were performed using a 40×/1.2 NA water objective (exc. eGFP and mCherry 480 nm and 560 nm, respectively, em. 520/35 nm and 600/50 nm). Images were scanned in 512×512 pixel format, with zoom factor of 4.0, and pixel size of 0.104 µm. Laser power was set to 400-4000 nW for the 480 nm laser and 200-2000 nW for the 560 nm laser. All other FLIM settings were identical as described above (FRET fluorescence lifetime imaging microscopy).

### 2.9 FRET-FLIM-based data analysis

SymPhoTime64 software was used to analyze the acquired FLIM data. For the analysis, the acquired data was rebinned two-fold from 512×512 pixel to 256×256 pixel. Additionally, a four-fold binning of the TCSPC resolution was used, *i.e.* 40 ps bin size. The first step of analysis was to select the ROI based on the fluorescence intensity in the green channel (**Supplementary Figure 1**). All pixel with less than 100 or more than 1200 photons were removed. Next, high intensity spots were removed manually. Based on the resulting ROI, two different types of analysis pipelines were followed: First, each pixel was analyzed separately (so-called pixelwise analysis), and second, the information from all pixels in the ROI was summed up (so-called group-wise analysis or ensemble analysis).

In the pixelwise analysis, first the FastFLIM option in the Picoquant software was used to estimate mean photon arrival time, $\tau_{FF}$ . For the next steps, we exported $\tau_{FF}$ , and the intensity images of both green and red channel as tif-files to further process them with Python scripts (**Supplementary Scripts 1** and **2**). For the intensity images, three different selections were defined: (*i*) green or donor intensity after donor excitation in the prompt window (0- 25 ns), $I^{D|D}$ , (*ii*) red or acceptor intensity after donor excitation in the prompt time window, $I^{A|D}$ , and (*iii*) acceptor intensity after acceptor excitation in the delay time window, $I^{A|A}$ (26-50 ns). These four images were converted into pixelwise tables and used to generate two-dimensional histograms.

Additionally, the total photon count of each signal within the ROI was summed to determine the total fluorescent signal intensities $I_{\Sigma }^{D|D}$ , $I_{\Sigma }^{A|D}$ , and $I_{\Sigma }^{A|A}$ , respectively. $I^{X|Y}$ is the photon count for detection at wavelength *X* after excitation at wavelength *Y*, and *Σ* indicates the summing of photons in all pixels.

The second approach was to analyze all pixels jointly. Here, a photon arrival time histogram from all green or donor photons inside the ROI was generated and decay fitting was performed by n-Exponential Reconvolution (n=2 or 3). We derived the species-weighted average lifetime $\tau_{avg}$ (29) of the donor in the absence (DO) or presence (DA) of the acceptor:


(3) $$\tau_{avg}=\;\sum_{i}x_{i}\tau_{i}\text{ with }\sum_{i}x_{i}=1$$

Where $x_{i}$ are the fractions of species *i*, and $\tau_{i}$ is the lifetime of species *i*.

The lifetime-based FRET efficiency $E_{\tau }$ was calculated by the donor lifetime of DO samples and DA samples (28):


(4) $$E_{\tau }=1-\frac{\tau_{avg,\;DA}}{\tau_{avg,\;DO}}$$

Finally, we used the exported intensity signal from both pixelwise and group-wise analysis to calculate the proximity ratio and stoichiometry. Note that both values were not corrected for crosstalk. The proximity ratio is given by (30):


(5) $$E_{PR}^{raw}=\frac{I^{A|D}}{I^{A|D}+I^{D|D}}$$

and the stoichiometry, which defines the composition of the formed receptor complex, is given by (30):


(6) $$S_{PIE}^{raw}=\frac{I^{D|D}+I^{A|D}}{I^{D|D}+I^{A|D}+I^{A|A}}$$

Additionally, the $S_{\Sigma PIE}^{raw}$ is given by the same equation with all $I^{X|Y}$ substituted by $I_{\Sigma }^{X|Y}$ . A stoichiometry value of 0 means no donor molecules in the complex, while a value of 1 means no acceptor molecules in the complex. A value of 0.5 represents an equal amount of donor and acceptor in the formed complex.

Total fluorescent signal intensity $I_{\text{Σ}}$ is given by:


(7) $$I_{\text{Σ}}=I^{D|D}+I^{A|D}+I^{A|A}$$

To consider the dependency of the FRET efficiency on the stoichiometry of the complexes, a dimerization grade was calculated based on the difference between each sample to the negative control normalized by the difference of the maximum FRET efficiency in positive control to that of the negative control.

The dimerization grade is given by:


(8) $$dimerization\;grade\;\left[\%\right]=100\times \frac{\bar{E_{\tau_{i}}(S_{\text{Σ}PIE}^{raw})}-\bar{E_{\tau_{\beta 1AR}}(S_{\text{Σ}PIE}^{raw})}}{\bar{E_{\tau_{CD28}}(S_{\text{Σ}PIE}^{raw}<0.3)}-\bar{E_{\tau_{\beta 1AR}}(S_{\text{Σ}PIE}^{raw})}}$$

Where the $E_{\tau_{i}}(S_{\text{Σ}PIE}^{raw})$ represents the FRET efficiency $E_{\tau }$ of *i* (Mc4r isoforms pairs) in the respective stoichiometry $S_{\text{Σ}PIE}^{raw}$ range, which is<0.3, 0.3-0.5, 0.5-0.7 and >0.7. Similarly, $E_{\tau_{\beta 1AR}}(S_{\text{Σ}PIE}^{raw})$ and $E_{\tau_{CD28}}(S_{\text{Σ}PIE}^{raw}<0.3)$ are the FRET efficiency $E_{\tau }$ of β1AR and CD28 at the indicated stoichiometry $S_{\text{Σ}PIE}^{raw}$ range.

### 2.10 Statistical analysis

Results are presented as mean ± SD unless otherwise stated in the figure legends. Statistical analyses were performed in Excel 2016 (Microsoft, Redmond, Washington, United States). Student’s unpaired *t*-test, with a two-tailed distribution, was performed on FRET-AB and FRET-FLIM results to compare each sample with negative control, and to compare donor/acceptor ratio 1:5 with corresponding donor/acceptor ratio 1:1. Significance was defined as *p*<0.05 in all cases.

## 3 Results

### 3.1 Fluorescent protein tagged receptors showed unaltered signaling

**Figure 2:**
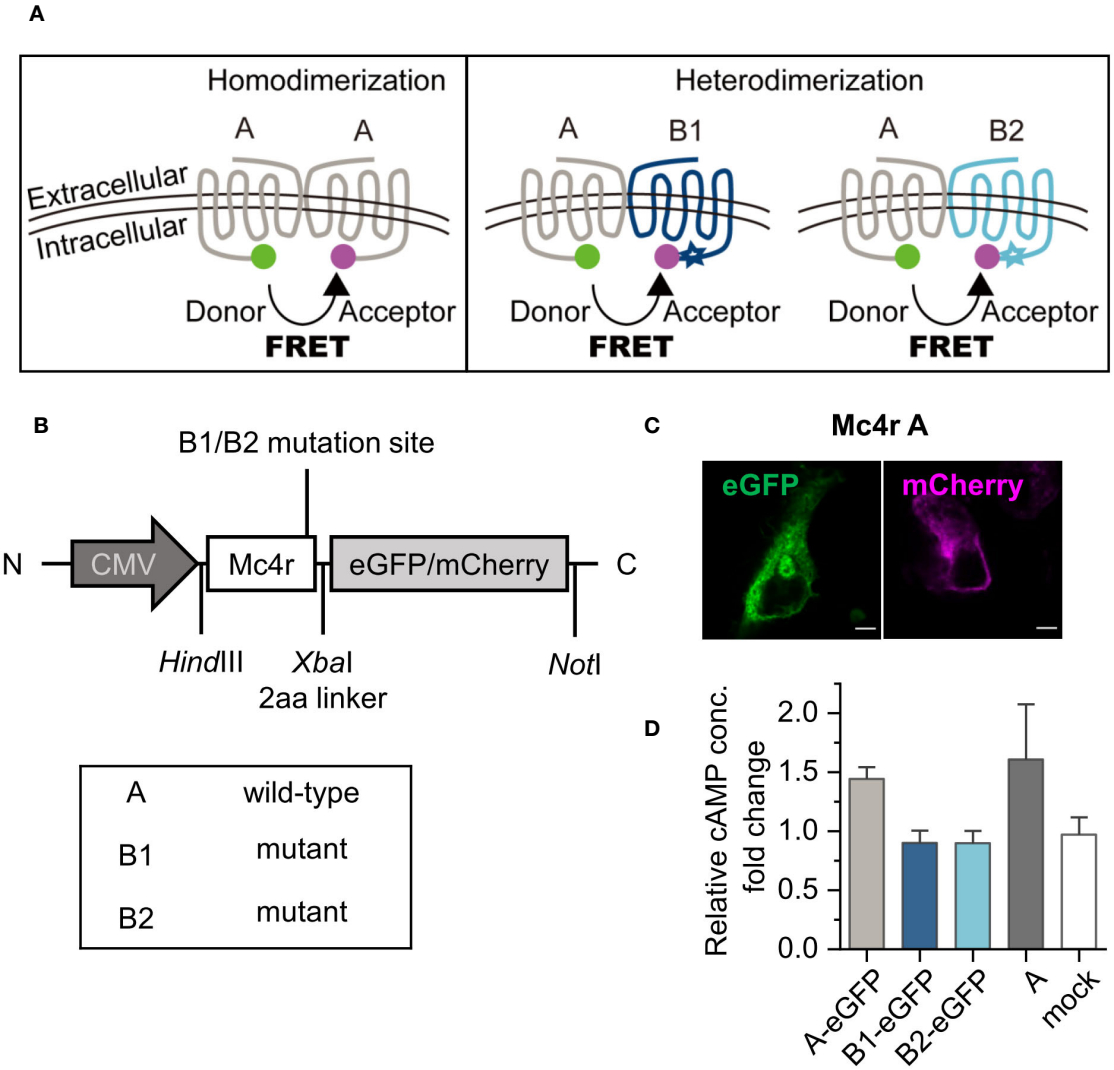
Dimerization hypothesis, experimental design, and constructs validation. **(A)** Puberty onset timing determination is hypothesized to function by receptor homodimerization or heterodimerization. In the scheme, receptors were color coded by grey for A, dark blue for B1 and light blue for B2. Stars indicate mutations at C-terminus. eGFP is used as donor, shown as a green sphere. mCherry is used as acceptor, shown as a magenta sphere. These receptor color schemes were used throughout the figures. **(B)** Constructs used in the study. eGFP or mCherry were fused to the C-terminus of Mc4r wild-type and mutant alleles. **(C)** Detection of expression of fusion proteins, here Mc4r A, by confocal microscopy. Scale bar: 5 µm. **(D)** cAMP assay of Mc4r A/B1/B2. Three fish Mc4r-eGFP constructs were used in the cAMP assay and compared to fish A constructs with no fluorescent protein. cAMP production from the samples without and with activation were measured with commercial cAMP kit. Details see in the methods. The fold changes of relative cAMP concentration were derived from cAMP concentration from one construct type with activation divided by that from the same construct without activation. A-eGFP can be activated by agonist similar to A constructs and B1/B2-eGFP are defective as expected. Data are from three independent experiments. All data are presented as mean ± SD.

Cell-based FRET measurements were performed to investigate the hypothesized receptor dimerization. The chosen pair eGFP/mCherry has shown sufficient spectral overlap (**Supplementary Figure 2**) to serve as donor/acceptor pair with a Förster radius of 52.4 Å (31) (**Figure 2A**).

The cloned fusion proteins carry eGFP or mCherry at the intracellular C-terminus of the receptors (**Figure 2B**). All receptor fusion proteins showed expression on the cell membrane (**Figure 2C**), and co-expression of two Mc4r isoforms showed correct cell surface expression (**Supplementary Figure 3**). The signal transducing activities of the recombinant receptors with the fluorophore tag were confirmed by measuring ligand-induced cAMP concentrations, using Mc4r A, B1, and B2 with eGFP *vs.* Mc4r A without tag. Constitutive activity and NDP-α-MSH induced activity [100 nM NDP-α-MSH is the saturation level in dose-response curves (10)] were compared for each Mc4r type. Mc4rA-eGFP showed a similar trend compared to untagged Mc4rA, while Mc4rB1-eGFP and Mc4rB2-eGFP cannot be stimulated (**Figure 2D**). This agrees with previous results for the untagged Mc4rB variants (5).

### 3.2 FRET-FLIM on fish Mc4r homodimer pairs revealed moderate A-A dimers formation

**Figure 3:**
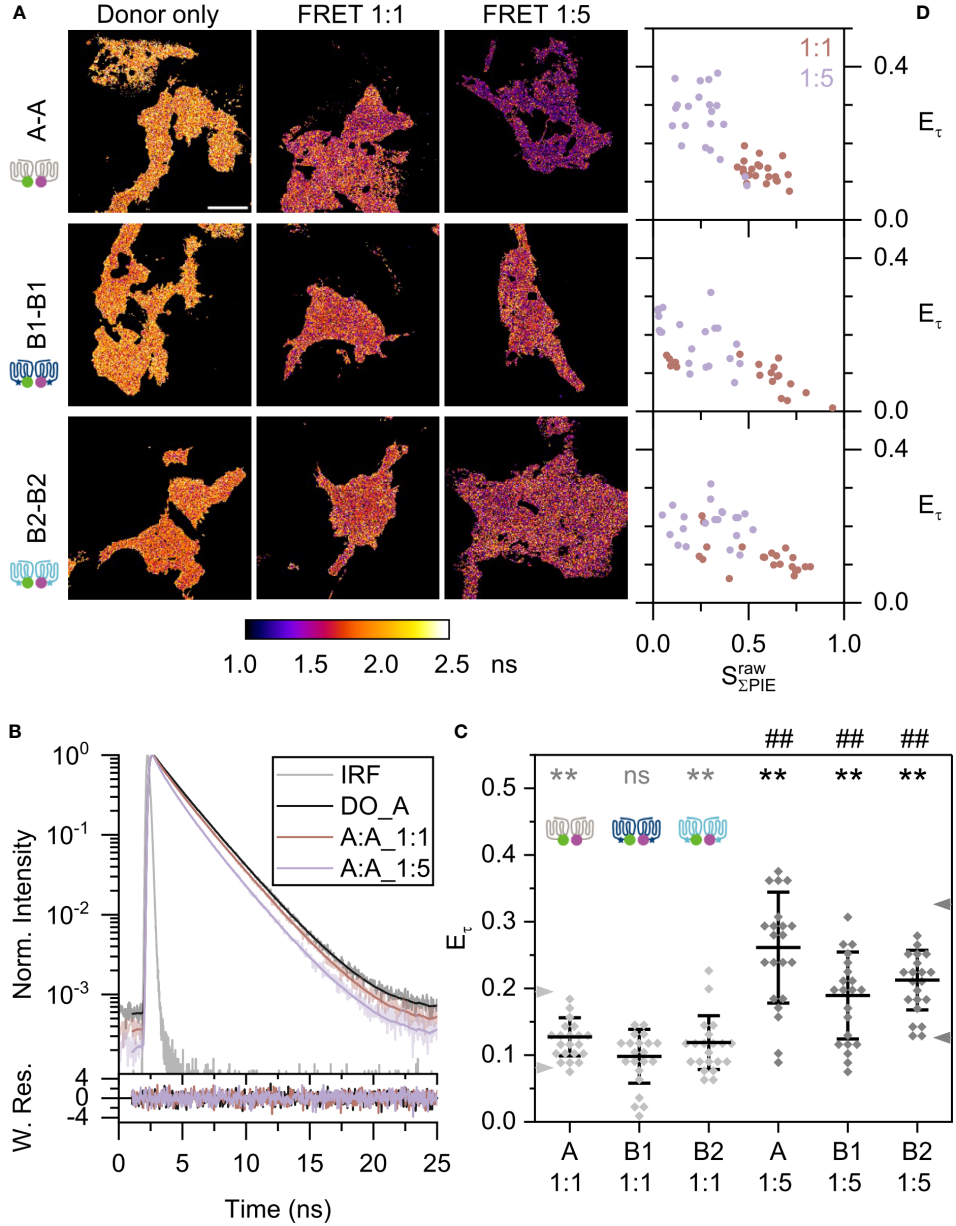
Fish Mc4r A-A, B1-B1, and B2-B2 homodimerization. **(A)** Species-weighted average lifetime of eGFP are displayed in the lifetime images. The lifetime range were set to 1.0 to 2.5 ns for all images in ImageJ. Scale bar: 10 µm. **(B)** Curve fitting by n-Exponential Reconvolution (n=2 or 3 exponential components), using data of one A-A pair as example in upper panel. Maximum intensity of donor-only, FRET 1:1 and FRET 1:5 are normalized to one. Grey curve is instrument response function, black curve is the donor-only, dark orange is A-A 1:1, and light purple is A-A 1:5. This color scheme is kept throughout the figures. The lower panel represents the weighted residues. Here is ensemble analysis as depicted in **Supplementary Figure 1**. **(C)** Scatter plot of lifetime-based FRET efficiency $E_{\tau }$ . Two arrowheads on the left axis are the reference lines of monomer and dimer controls at 1:1 ratio; and on the right axis from 1:5 ratio. The overall mean (horizontal bar), and standard deviation of the mean (error bars) are indicated. Two tails unpaired *t*-test are used in statistical analysis. Grey *: compared to β1AR 1:1. Black *: compared to β1AR 1:5. Black #: 1:1 *vs.* 1:5 for each pair. Statistics (summary in **Supplementary Table 2**): ns: not significant, **/##: *p*<0.01. **(D)** 2D scatter plots of lifetime-based FRET efficiency to effective stoichiometry for all Mc4r homodimers. FRET samples of donor/acceptor ratio 1:1 is shown in dark orange and ratio 1:5 in light purple.

We first studied the homodimerization of Mc4r receptor isoforms. Total fluorescent signal intensity $I_{\text{Σ}}$ of all cells and all protomer combinations were distributed within one order of magnitude (2-15×10^6^), thus allowing us to compare among cells and protomer combinations (**Supplementary Figure 4**). To establish a reference for the expected FRET efficiencies, defined monomer (β1AR) and dimer (CD28) controls were measured (**Supplementary Figure 5**). β1AR at 1:1 and 1:5 ratios showed a minimum reduction of lifetime in comparison to donor-only reference, which did not depend on the expression levels as judged by total photon countrate. On the other hand, lifetime of CD28 was clearly reduced at both 1:1 and 1:5 ratios (**Supplementary Figure 6A**). If similar FRET occurs between the different Mc4rs, a donor lifetime decrease should be observed in the presence of the acceptor. This is indeed seen clearly in the Mc4r pairs A-A and is also relevant for B1-B1 and B2-B2. **Figure 3A** shows the average lifetime based on pixelwise calculation of the species-weighted lifetimes [equation (3)]. Color scale of lifetime images corresponds to average lifetime with light orange being longest. A-A pair showed a decrease of donor lifetime from 2.13 ns to 1.85 ns and 1.57 ns, respectively, when going from donor-only to FRET 1:1 and FRET 1:5 ratio samples (**Supplementary Figure 6B**).

Time-resolved fluorescence intensities were analyzed here by ensemble analysis (see **Supplementary Figure 1**) and were compared between the FRET samples and those of the donor-only reference. The slope of the decay provided a measure of donor quenching and depended on transfection ratio. For the Mc4r A-A sample, the slope was steeper than for the A donor-only sample (**Figure 3B**). The steeper slope at a 1:5 ratio than at a 1:1 ratio corresponded to a higher proportion of quenched donor and a lower average lifetime. A more meaningful parameter to compare the time-resolved FRET-induced donor decay is the measure of FRET efficiency [equation (4)], which rises along with more donor quenching. Monomer reference β1AR had FRET efficiencies $E_{\tau }$ of 8.1 ± 4.3% for 1:1 and 12.6 ± 3.8% for 1:5 ratios; dimer reference CD28 had FRET efficiencies $E_{\tau }$ of 19.5 ± 5.1% for 1:1 and 32.6 ± 5.7% for 1:5 ratios (**Supplementary Figure 5B**). These provide two reference lines, which serve as lower and upper boundaries for the two oligomerization states (monomer and dimer) and are distinct for the 1:1 and 1:5 ratios.

All Mc4r pairs A-A, B1-B1, and B2-B2 showed FRET efficiencies between the two reference lines. Among them, A-A has highest FRET efficiencies $E_{\tau }$ of 12.7 ± 2.9% for 1:1 and 26.1 ± 8.3% for 1:5 ratios (**Figure 3C**). This is an up to two-fold increase compared to β1AR (*p*<0.01). In contrast, B2-B2 displayed lower FRET efficiencies $E_{\tau }$ of 11.9 ± 4% for 1:1 and 21.2 ± 4.5% for 1:5 ratios, followed by B1-B1 with $E_{\tau }$ of 9.8 ± 4% for 1:1 and 18.9 ± 6.5% for 1:5 ratios. They were significantly higher than for β1AR (*p*<0.01) except for the B1-B1 1:1 ratio. Comparing FRET efficiencies (average values) from three biological replicates reveals a random distribution of FRET efficiencies as expected (**Supplementary Figure 7A**).

Furthermore, an important parameter to visualize the concentration dependency of dimerization is stoichiometry. The stoichiometry is defined as ratio of donor fluorophores by all the fluorophores molecules in the complex. Here the complex might be monomer or dimer or higher-order oligomers. According to equation (6) a $S_{PIE}^{raw}$ close to 1 indicates a substantial donor-only fraction among the complexes, while close to 0 states for a substantial acceptor-only fraction. In combination with the FRET efficiency as a readout, it gives a hint to which degree dimerization occurs between two binding partners. In the 2D scatter plot $S_{\Sigma PIE}^{raw}\;$ *vs.* $E_{\tau }$ , β1AR reflects small changes of FRET efficiencies along with stoichiometry that gives a hint whether FRET occurs due to random collisions (**Supplementary Figure 5C**). On the contrary, FRET efficiency of CD28 was considerably raising with descending stoichiometry including a trend toward saturation. This indicates a clear interaction of two binding partners and complex formation.

Applied to A-A, B1-B1, and B2-B2, all showed an increasing FRET efficiency with drop down of stoichiometry (**Figure 3D**). Among the three, A-A still displayed the highest growth within a certain range of stoichiometry and the other two a moderate increase. In conclusion, Mc4r isoforms form homodimers. Especially A-A is highly favorable in dimerization.

Of note, such investigations assume that donor-only probes are treated the same way like donor-acceptor probes. To get a convincing proof, FRET acceptor photobleaching (FRET-AB) was applied on the same samples. FRET-AB measurements show similar trends like FRET-FLIM, but show smaller changes (**Supplementary Figures 8A, B**), most likely due to various competing effects such as donor photo-destruction during acceptor depletion, incomplete bleaching of acceptors, and generation of fluorescent photoproducts (32).

### 3.3 FRET-FLIM on fish Mc4r heterodimer pairs revealed A-B1 and A-B2 dimers

**Figure 4:**
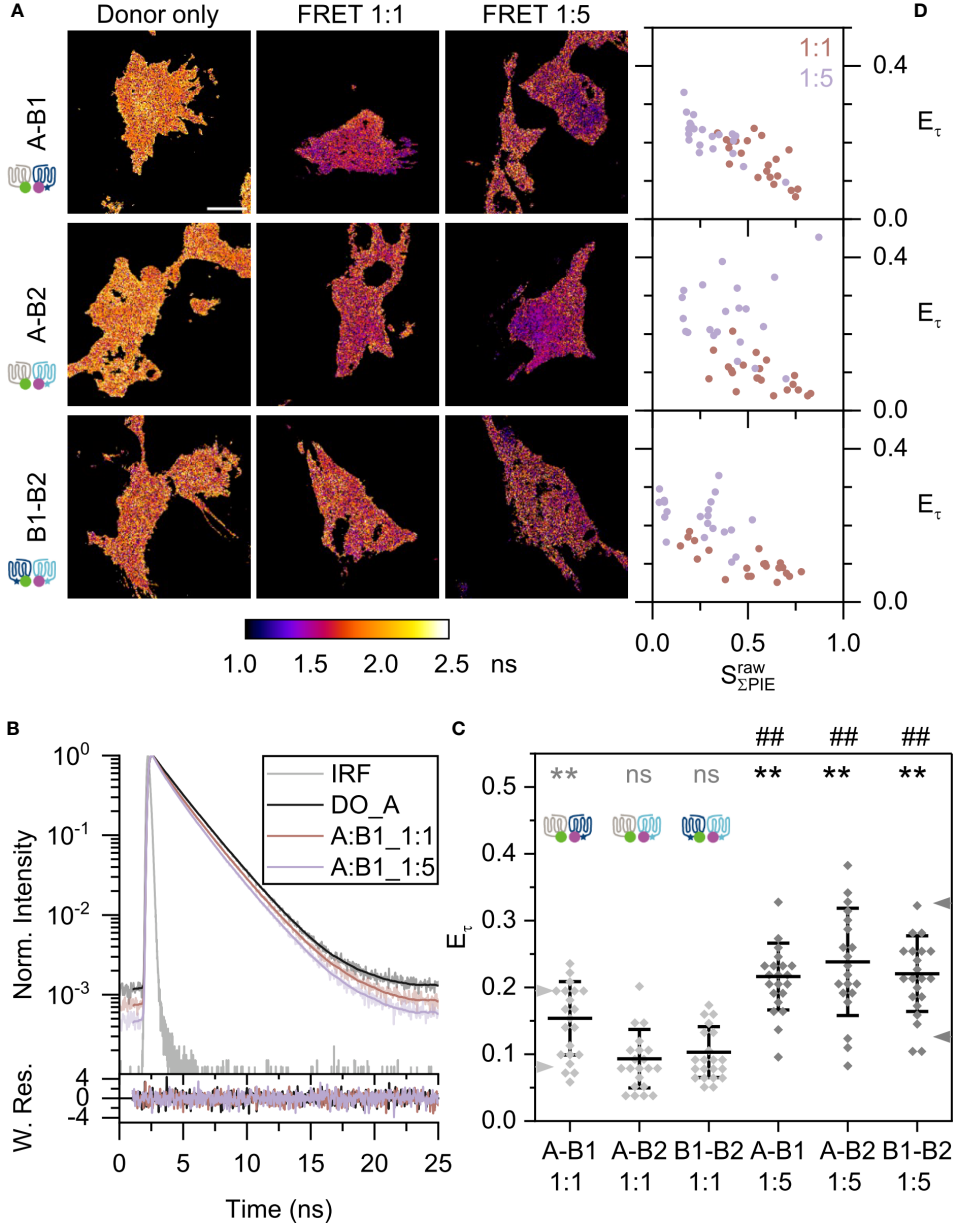
Fish Mc4r A-B1, A-B2, and B1-B2 heterodimerization. Each panel is presented in the same way as in **Figure 3**. **(A)** Lifetime images show species-weighted average lifetime of eGFP. Scale bar: 10 µm. **(B)** Curve fitting, using data of one A-B1 pair as example in upper panel. The lower panel shows the weighted residues. **(C)** Scatter plot of lifetime-based FRET efficiency $E_{\tau }$ . Statistics (summary in **Supplementary Table 2**): ns: not significant, **/##: *p*<0.01. **(D)** 2D scatter plots of lifetime-based FRET efficiency to effective stoichiometry for all Mc4r heterodimers.

Next, we analyzed the heterodimerization of Mc4r receptor isoforms using the same setup and scheme as described above. Thus, combinations of Mc4r A-B1, A-B2, and B1-B2 were investigated. The average donor-only lifetime was roughly 2 ns and consistent over all measurements (**Supplementary Figure 6C**). The lifetime images showed that the donor lifetime is getting shorter with increasing amounts of acceptor (**Figure 4A**). The steeper slope of the time-resolved fluorescence intensities of the Mc4r A-B1 sample (**Figure 4B**) also proves this fact. According to the reduced average fluorescence lifetime, A-B1 revealed highest FRET efficiencies $E_{\tau }$ of 15.4 ± 5.5% for 1:1 and 21.6 ± 5% for 1:5 ratios among all mixed isoforms (**Figure 4C**). On the other hand, both A-B2 and B1-B2 showed a similar trend. A-B2 had FRET efficiencies $E_{\tau }$ of 9.3 ± 4.4% for 1:1 and 23.8 ± 8% for 1:5 ratios; and B1-B2 displayed FRET efficiencies $E_{\tau }$ of 10.3 ± 3.8% for 1:1 and 22.1 ± 5.7% for 1:5 ratios. The $E_{\tau }$ of samples with a 1:5 ratio was significantly higher than those for β1AR (*p*<0.01). A significant heterodimerization for A-B2 and B1-B2 at 1:1 ratio was not observed. Looking at variation among replicates, only a minor variation was observed (**Supplementary Figure 7B**).

In the 2D scatter plot $S_{\Sigma PIE}^{raw}\;$ *vs.* $E_{\tau }$ , A-B1 and A-B2 showed a clear raising FRET efficiency with decrease of stoichiometry; A-B2 exhibited a wider distribution (**Figure 4D**). In comparison, B1-B2 showed the slowest increase of FRET efficiency.

In light of the fact that the interaction between the two protomers A and B1 can be in a reversed order (A-B1 and B1-A), it is interesting to know to which direction the equilibrium moves. It is noteworthy to mention that expressions of A and B1 are high in the large males, supporting its important role. Donor lifetime of B1-A changes like A-B1 in a same manner for 1:5 ratio, with FRET efficiencies $E_{\tau }$ of 7.9 ± 3.6% for 1:1 and 21.2 ± 6.8% for 1:5 ratios (**Supplementary Figures 9A, B**). In the 2D scatter plot $S_{\Sigma PIE}^{raw}\;$ *vs.* $E_{\tau }$ , B1-A showed an increased FRET efficiency with steepness smaller than A-B1 (**Supplementary Figure 9C**). This indicates a different equilibrium in B1-A compared to A-B1, which may come from the fraction of high-FRET A-A homodimers and low-FRET B1-B1 homodimers in the mix (**Figure 3**).

In addition, FRET-AB was applied on the same samples. Similar as in the FRET-FLIM approach, A-B1 was observed to have the highest tendency to form heterodimers (**Supplementary Figure 8C**). In summary, Mc4r isoforms do form heterodimers, and A-B1 and A-B2 are the preferred ones among the tested heterodimers.

### 3.4 Agonist activation does not affect Mc4r dimerization

**Figure 5:**
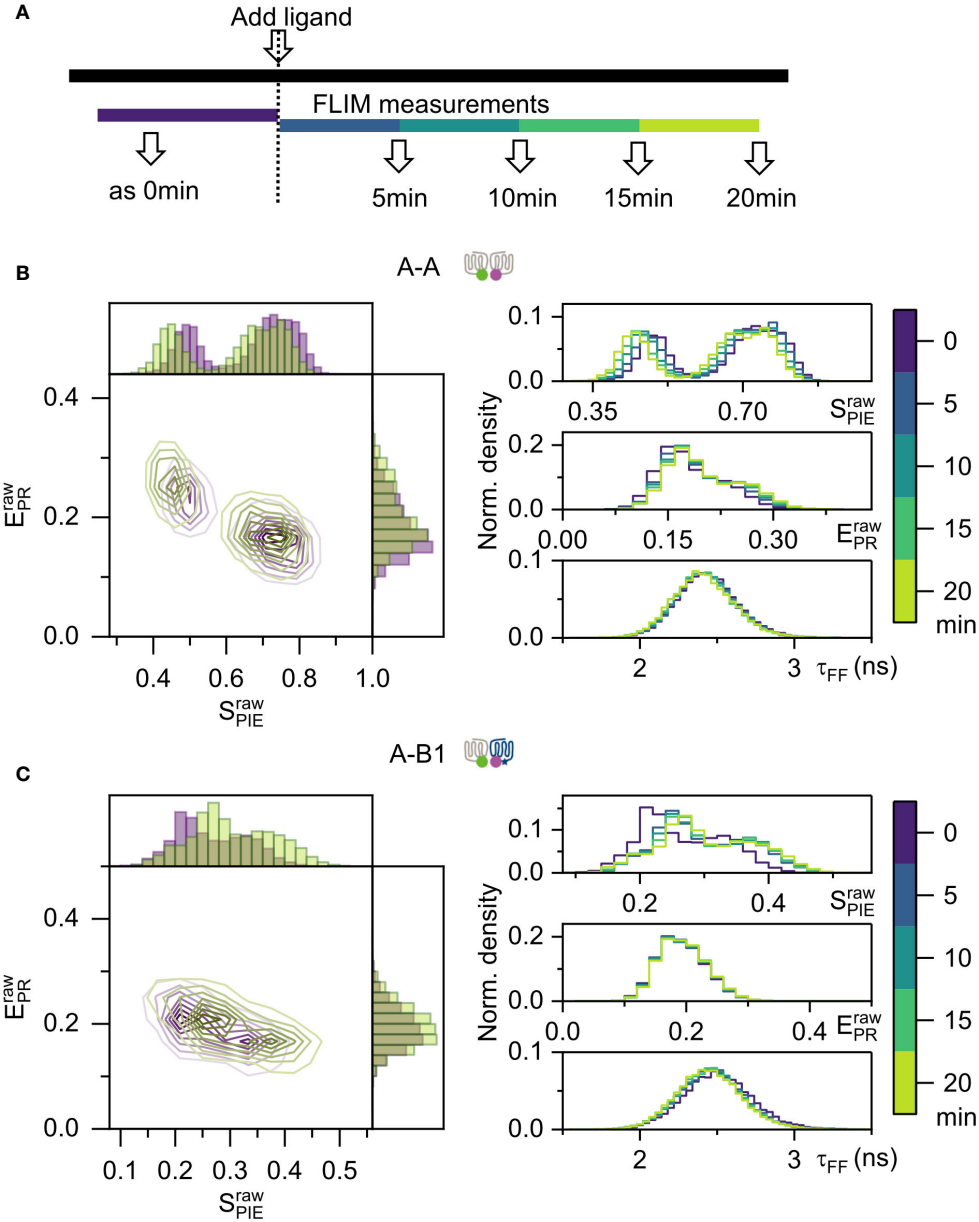
FRET-FLIM measurement on live cells, activated by NDP-α-MSH. **(A)** Activation and measurement scheme. One FLIM measurement were performed before adding the ligand. After applying ligands, FLIM measurement were performed at 5 min, 10 min, 15 min, and 20 min post- adding. **(B, C)** Proximity ratio $E_{PR}^{raw}$ and stoichiometry $S_{PIE}^{raw}$ were calculated by equation (5) and (6), from individual cells. One representative cell example was used in the pixelwise analysis. On the left panel, contour color maps were displayed with 8 levels in a linear scale, showing 0 min in purple and 20 min in light green. Histogram were plotted with 50 bins for both axes. On the right panel, histograms display changes over time. Here is pixelwise analysis as depicted in **Supplementary Figure 1**. **(B)** Samples A-A with donor/acceptor ratio 1:1. **(C)** Samples A-B1 with donor/acceptor ratio 1:1.

It has been reported that ligands can influence GPCR dimerization (33, 34). We asked if agonist activation with NDP-α-MSH has an influence on the dimerization of Mc4r A-A and A-B1. NDP-α-MSH is a highly potent synthetic agonist analog to natural ligand α-MSH with an EC50 of 4.00 nM for A-A and 6.36 nM for A-B1 (10). Based on this information, a concentration of 100 nM (saturation level) was picked for the full activation of Mc4r. For each cell, five measurements were taken (**Figure 5A**). Minor changes were observed over time. For the A-A 1:1 ratio, $S_{\Sigma PIE}^{raw}$ showed a slight decrease while $E_{\tau }$ slightly increased (**Supplementary Figure 10A**). In case of the A-B1 1:1 ratio, $S_{\Sigma PIE}^{raw}$ and $E_{\tau }$ both slightly increased (**Supplementary Figure 10B**). To avoid that heterogeneity is averaged out and reveal the variations within single cells (35), a pixelwise calculation of proximity ratio $E_{PR}^{raw}\;$ and stoichiometry $S_{PIE}^{raw}$ was applied using equation (5) and (6) (refer to **Supplementary Figure 1**). Here the histogram of $E_{PR}^{raw}$ was mostly unaltered while the histogram of $S_{PIE}^{raw}$ showed a minor shift towards lower values for A-A and higher values for A-B1 (**Figures 5B, C**). Mean photon arrival time $\tau_{FF}$ was also not changed. This leads to the conclusion that agonist NDP-α-MSH does not substantially influence the dimerization state of A-A and A-B1.

### 3.5 Mc4r orthologues showed similar dimerization in fish and human

Previous studies of human MC4R orthologues showed that MC4R wild-type (WT) dimerizes and chimeric MC4R chim7 (named Mut in this study) has reduced dimerization capacities (20, 27). To compare the dimerization capacity of the fish to the human receptors, similar experiments were applied to human MC4R WT and Mut. Donor lifetime was clearly shorter with a higher amount of acceptor in WT-WT and WT-Mut and the effect was less pronounced in Mut-Mut (**Supplementary Figures 11A, B**).

Among the three, WT-WT showed highest FRET efficiency $E_{\tau }$ of 10.2 ± 5.1% for 1:1 and 23.6 ± 6.4% for 1:5 ratios, which is less than but close to A-A (**Supplementary Figure 11C**). Mut-Mut displayed lowest FRET efficiency $E_{\tau }$ of 7.5 ± 4.8% for 1:1 and 13.8 ± 7.4% for 1:5 ratios being indistinguishable from β1AR, indicating it is a monomer. WT-Mut with $E_{\tau }$ of 7.4 ± 3.3% for 1:1 and 19.6 ± 7.4% for 1:5 ratios showed a high FRET efficiency at 1:5 ratio. Compared to A-B1 1:5 ratio which is 1.72-fold higher than β1AR, WT-Mut has a 1.56-fold change. This demonstrates that the dimerization capacity of the human WT is highly similar to that of fish A representing the fish wild-type Mc4r version. FRET-AB with the same samples (WT-WT, Mut-Mut, WT-Mut) showed a similar trend with random distribution among their replicates (**Supplementary Figure 11D**).

### 3.6 Dimerization grade is high for A-A, A-B1 and A-B2

Since the range of donor/acceptor ratio is limited by the level of expression in the transfected cells in the experimental scheme, all stoichiometry values were categorized into four ranges<0.3, 0.3-0.5, 0.5-0.7, and >0.7 and a dimerization grade was calculated according to equation (8). Note the FRET efficiency depends on the stoichiometry and it is expected that lower stoichiometry, thus higher amount of acceptor molecules in the complex, corresponds to higher FRET efficiency. The calculation of dimerization grade was based on the following two assumptions: (1) FRET occurring in the β1AR measurements is caused by random collisions of receptors; thus, representing the monomer baseline. (2) The maximum FRET determined from CD28 gives the 100% dimerization line; this level is supposed to be reached at a certain protein concentration by all other receptors.

**Figure 6:**
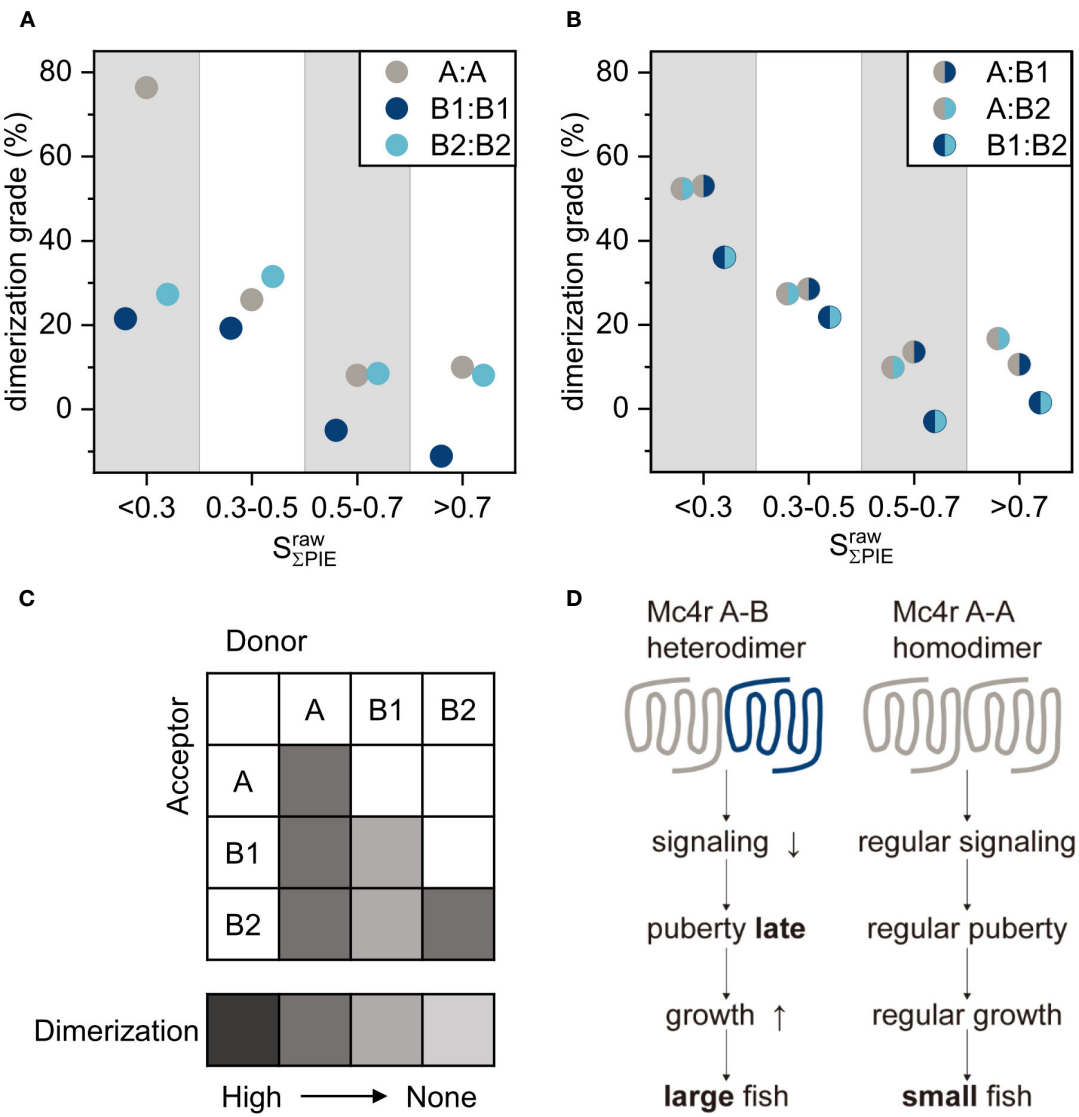
Dimerization grade summary and model of Mc4r interaction in fish. **(A, B)** Dimerization grade of dimers in four stoichiometry categories (<0.3, 0.3-0.5, 0.5-0.7, >0.7), calculated by equation (8). **(A)** Fish homodimers A-A, B1-B1, B2-B2. **(B)** Fish heterodimers A-B1, A-B2, B1-B2. **(C)** Summary of dimerization tendency of all interaction partners, as depicted by color code with dark grey being the highest. The white squares were not determined. A-A and A-B1/B2 are the most prominent dimers. **(D)** Model of molecular basis of differential Mc4r puberty signaling. Mc4r A-A homodimers have regular signaling leading to early puberty and growth, and these males mature as small fish. Mc4r A-B heterodimers have reduced signaling leading to late puberty and continued growth, and thus these males mature as large fish.

FRET efficiency of CD28 at stoichiometry of<0.3 reached a saturation level with $E_{\tau }$ of 34.1%. This is taken as 100% dimerization. A-A had a 26% dimerization grade at 0.3-0.5 stoichiometry and increased further to 76% at<0.3 (**Figure 6A**). B1-B1 had around 20% dimerization grade at both 0.3-0.5 and<0.3 stoichiometry. B2-B2 reached a level of 30% for both stoichiometry ranges. Both A-B1 and A-B2 had about 28% and 52% dimerization grade for 0.3-0.5 and<0.3 stoichiometry, respectively (**Figure 6B**). B1-B2 showed low dimerization grade of roughly 22% for 0.3-0.5 and increased to 36% for<0.3 stoichiometry. This leads to the conclusion that the dimerization grade for A-A, A-B1, A-B2 and B2-B2 is moderate, while on the lower side for B1-B1 and B2-B2 (**Figure 6C**).

## 4 Discussion

Here, we investigated how on the molecular level the genetic polymorphism of Mc4r alleles determines the onset of puberty in *Xiphophorus*. Although a dominant negative effect of mutated receptor isoforms on the wild-type receptor in heterodimers has been postulated, there has been no direct evidence at the molecular level that different Mc4r alleles undergo receptor dimerization. We addressed this problem in a fluorescence-based assay by using eGFP/mCherry tagged Mc4r in FRET-based experiments and showed that: (1) Mc4r A-A form homodimers; (2) Mc4r A-B1 and A-B2 form heterodimers; and (3) NDP-α-MSH activation does not influence A-A and A-B1 dimerization.

### 4.1 The A isoform forms A-A homodimers and has high cAMP signaling

Different from class C GPCRs, for which dimerization is proved to be essential for their full function (36); there is still debate about the dimerization of class A GPCRs (37, 38). This question concerns several aspects, including the existence of dimers, to which extent they dimerize, the structural change induced by the dimerization and the functionality of the dimerization. In our study we show for the *X. nigrensis* and *X. multilinearus* Mc4r, a class A GPCR, homodimerization of Mc4r A isoforms. The dimerization of fish Mc4r A is in agreement with a study on the human MC4R WT receptor (20), and we confirmed these findings in our FRET-FLIM measurements. Like the human MC4R WT, *Xiphophorus* Mc4r A is also a wild-type receptor, being the “default” functional receptor type in mediating the downstream signaling. As we expressed a fish receptor in a mammalian cell, we cannot fully exclude that the degree of dimerization is higher in a fish cell if the membrane composition differs.

Combining the results of FRET-FLIM and the cAMP assay clearly shows that in A-A homodimers there is no change of dimerization and still high cAMP production upon agonist activation. In some other class A GPCRs, effect of ligand binding on dimerization was reported. Basal μOR dimers are lowly abundant but agonist DAMGO activation induces μOR dimerization (34). CXCR4 forms transient homodimers, and the agonist CXCL12 does not alter dimeric stoichiometry, but several inverse agonists disrupted the dimers by inhibiting constitutive activity (33). Thus, this indicates a different molecular basis of dimerization among different GPCRs. Mc4r constitutive activity is inhibited by inverse agonist Agrp, which could be an additional candidate ligand to apply on Mc4r dimers to better understand its pharmacology, though this is beyond the scope of the current study.

### 4.2 B preferentially forms dimers with A

Comparable to A forming A-A homodimers, non-functional mutant Mc4r isoforms B1 and B2 can also form homodimers and B1-B2 heterodimers, but to a lesser degree. Most importantly, both B1 and B2 form heterodimers with A in an effective manner. In one human MC4R study, heterodimerization of the WT MC4R with a mutant receptor D90N was shown, suggesting a dominant negative effect of the D90N mutation (20). This effect is quite similar to what we observed for the mutant B isoform on the wild-type Mc4r A in fish.

Furthermore, the analysis of the dimerization grade showed A-B1 and A-B2 have reached 28% dimerization for stoichiometry 0.3-0.5 and even higher for <0.3. This gives several insights into the A-B interaction. First, B competes with A in the equilibrium so that a high fraction of A-B is formed. Second, A-A fraction is low in a mixture of A, B, A-A, B-B, and A-B, and largely reduced when the amount of B is increasing, supported by the increase of FRET in case of stoichiometry <0.3. Third, B-B is less present already at 0.3-0.5 stoichiometry, shown by a saturation level at 0.3-0.5 and <0.3. Together, these results demonstrate that the A-B heterodimer constitutes the main receptor population when cells express both A and B type receptors.

To integrate the results from the *in vitro* cell culture assay into the physiology of fish for inferring the molecular interactions in the fish brain *in vivo*, several lines of evidence need to be considered. First, A and B1 alleles are expressed at equally high levels in large male fish brain (10). This is correlated to the stoichiometry level of about 0.5. Second, A and B isoforms differ only in the most distal part of the C-terminus. Thus, it is most likely that the interaction interface is not affected, and the dimerization propensity depends primarily upon receptor concentration, thus affinity. Third, constitutive activity is similar between A-A and A-B1 (10). Along with the evidence of dimers in the absence of ligand activation, it suggests that a basal signaling activity is due to constitutive dimer formation. Fourth, binding of agonist has low on- and off-rates. As shown previously in a kinetic study (39), association half-time of [^125^I]NDP-MSH (0.23 nM) to Mc4r was 19 ± 1 min, and dissociation of same ligand reached a plateau at 50 ± 4% with half-time of 80 ± 15 min. We showed no change of dimerization upon activation for at least 20 min. This is in agreement with a role of Mc4r as a neuropeptide receptor on energy homeostasis with a slow-onset, long-lasting modulation (40). These evidences support the Mc4r dimerization as the molecular mechanism in fish brain to induce differential puberty signaling.

Of note, B1-A showed a lower FRET efficiency than A-B1 due to the receptor affinity difference. In cells expressing both A and B1, there are several components in the dimerization equilibrium that have to be considered: the monomers A and B, and four different dimer compositions (A-A, B1-B1, A-B1 and B1-A). The different receptor affinities determined from homodimerization experiments lead to a proposed competing equilibrium of the different receptor monomers and dimers. Since A-A is more prone to dimerize than B1-B1, the remaining B1 monomer concentration is likely higher than that of A. As a result, in the mixture, the amount of remaining donor protomers may be higher for B1-eGFP/A-mCherry, than for A-eGFP/B1-mCherry, and the B1-eGFP is less quenched by A-mCherry.

### 4.3 Mc4r homo- and hetero-dimerization mediates differential puberty signaling

The physiological consequence of our molecular findings is that Mc4r homo- and hetero-dimerization mediates differential puberty signaling of small and large males (**Figure 6D**). In small males, Mc4r A forms A-A homodimers due to absence or low levels of B isoforms while cAMP signaling is normal. It reaches a threshold level relatively early in development, leading to an early onset of puberty while the fish have grown to only a small size. In large males, Mc4r A is preferentially recruited to form A-B1 and A-B2 heterodimers, where cAMP signaling is defective so that the threshold is reached at a later time, thus leading to late puberty onset timing and maturity when the fish have grown to already a large size. In evolutionary terms, this means that *Xiphophorus* fish made use of a defective, malfunctioning allele, the signaling-incompetent B isoforms, to evolve an adaptive trait.

Genetic studies have shown that small males have either no or only few copies of B, while large males have many B copies and express a lot of B1 (5, 10). Mc4r A-A dimerization is more dominant in signal transduction in small males. On the other hand, Mc4r A-B1 dimerization is presumably more prevalent and essential in signaling in large males. Our results explain how the defective B1 receptor acts on the functional wild-type A receptor in a heterodimer and modifies the signaling strength of A allele encoded receptors in those fish that carry more B alleles than those with a low number of B alleles or no B allele at all.

It is clear that Mc4r is one important factor mediating energy intake conserved from fish to human. Here, we explain the molecular basis in *X. nigrensis* and *X. multilineatus* fish, where regulation of the puberty onset is mediated by a dominant negative effect of defective B alleles on A alleles, via Mc4r heterodimerization. Mutations of Mc4r from A to B alleles in males are preserved through selection pressure, because the large male phenotype is well suited for survival in nature. Other fish species, such as cave populations of the Mexican cavefish, *Astyanax mexicanus*, show mutations in Mc4r (41) as a cause of adaptation to the nutrient-poor cave environment. Reduced basal activity and reduced maximal response of such mutated receptors induce an elevated fish appetite, growth, and starvation resistance. In the light of these characteristics, our findings give molecular background to connect energy balance and reproduction. Interestingly, a closely related melanocortin receptor, MC3R, has been reported to be important for regulating onset of puberty in human. Humans carrying loss-of-function mutations in *MC3R* show delayed onset of puberty (42), and *MC3R* mutations are overrepresented in patients with constitutional delay of growth and puberty (43). Hence, this additionally underlines the dual role of melanocortin receptors MC4R and MC3R in controlling both food intake and influencing puberty onset timing across species.

## 5 Conclusion

In summary, our study demonstrates that Mc4r’s of *Xiphophorus* can form homo- and heterodimers. Such dimerization provides an explanation of how differential puberty signaling is mediated in small and large males of *X. nigrensis* and *X. multilineatus* fish. Furthermore, direct evidence of dimerization from the animals will be ideal. Despite that many protein interaction questions have been explored by antibody-based assay like proximity ligation assay, it is not applicable here due to the absence of two antibodies with high specificity to recognize the two highly identical protein isoforms. Thus, further investigations may be required on transgenic model fish to verify the interaction *in vivo* in fish brain in the future. Nevertheless, our work proves that Mc4r as a class A GPCR forms dimers and furthermore that these dimers are of physiological and regulatory value. This is not only important for *Xiphophorus* fish, but is also relevant for understanding metabolic regulation in other fish species and even metabolism and obesity in human.

## Data availability statement

The raw data supporting the conclusions of this article will be made available by the authors, without undue reservation.

## Ethics statement

Ethical approval was not required for the studies on humans in accordance with the local legislation and institutional requirements because only commercially available established cell lines were used. Ethical approval was not required for the studies on animals in accordance with the local legislation and institutional requirements because only commercially available established cell lines were used.

## Author contributions

RL: Formal Analysis, Funding acquisition, Investigation, Visualization, Writing – original draft, Writing – review & editing. MF: Formal Analysis, Investigation, Visualization, Writing – original draft, Writing – review & editing. KH: Formal Analysis, Writing – review & editing. KJ: Investigation, Writing – review & editing. MA: Writing – review & editing. MS: Conceptualization, Supervision, Writing – review & editing. KH: Conceptualization, Funding acquisition, Supervision, Writing – review & editing.
