# Supplementary material for: Dimerization of melanocortin 4 receptor controls puberty onset and body size polymorphism
Source: Front Endocrinol (Lausanne). 2023 Nov 10;14:1267590. doi: 10.3389/fendo.2023.1267590 (PMC10667928; doi:10.3389/fendo.2023.1267590)
Supplement: Supplementary file 1 [file DataSheet_1.pdf]

Supplementary Material

1 Supplementary Figures and Tables

1.1 Supplementary Figures

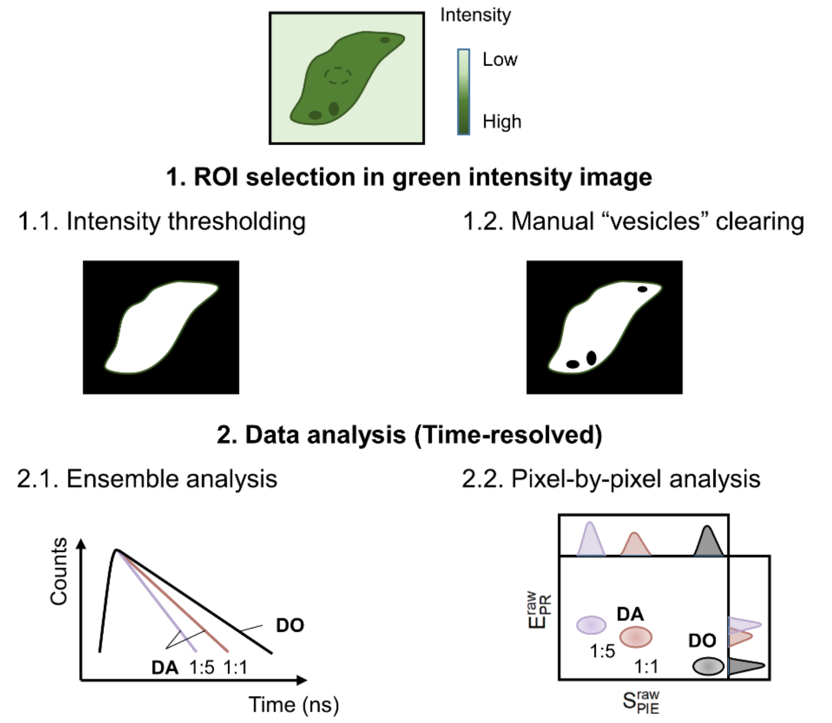

3. Parameter – readout

3.1. Ensemble analysis

	Parameter
$\tau_1, x_1, \tau_2, x_2, \ldots$	Lifetime and fraction of species 1,2,...
τ_{avg}	Species-weighted average lifetime
E_τ	Lifetime-based FRET efficiency
$I_\Sigma^{D\|D}, I_\Sigma^{A\|D}, I_\Sigma^{A\|A}$	Signal intensities sum of all pixels
$S_{\Sigma PIE}^{raw}$	Stoichiometry of summed intensity

3.2. Pixel-by-pixel analysis

	Parameter
τ_{FF}	Average photon arrival time
$I^{D\|D}, I^{A\|D}, I^{A\|A}$	Signal intensities
S_{PIE}^{raw}	Stoichiometry
E_{PR}^{raw}	Proximity ratio
I_Σ	Sum of signal intensities

Supplementary Figure 1. Schemes of PIE-based FRET-FLIM experimental design.

After ROI selection, two analysis pipelines were performed: ensemble analysis and pixelwise analysis. PIE mode allows for retrieving stoichiometry information. The ensemble approach concludes the dimerization based on overall changes of donor lifetime and stoichiometry. In the pixelwise approach, the parameters such as donor lifetime and stoichiometry are calculated per pixel and displayed as one- or two-dimensional distributions. Here, the ensemble approach robustly provides information of multiple lifetime species while the pixelwise approach was only used to reveal heterogeneity in live-cell agonist activation experiment.

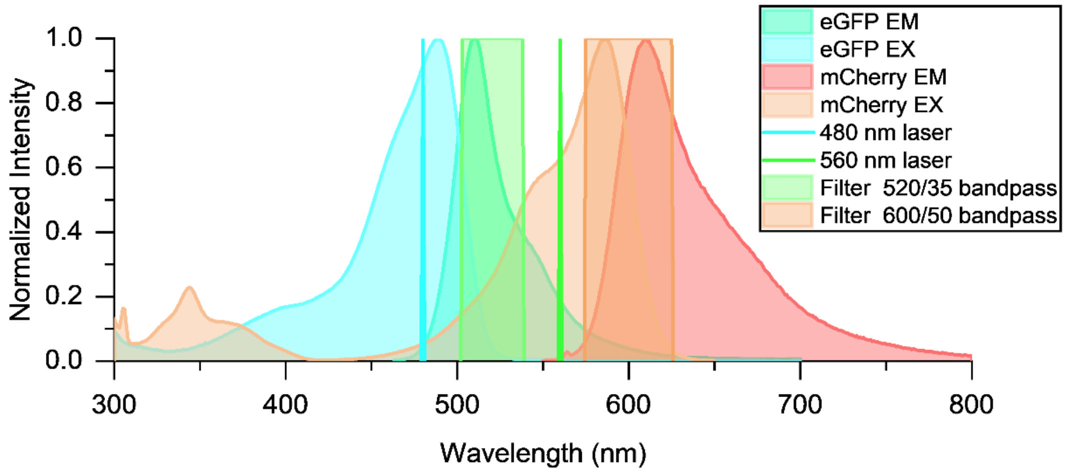

Supplementary Figure 2. Spectral information of fluorescent proteins used in FRET.

Excitation and emission spectra of eGFP and mCherry. Laser lines (480 nm, 560 nm) and filters (520/35 and 600/50) used in the FLIM measurement were indicated.

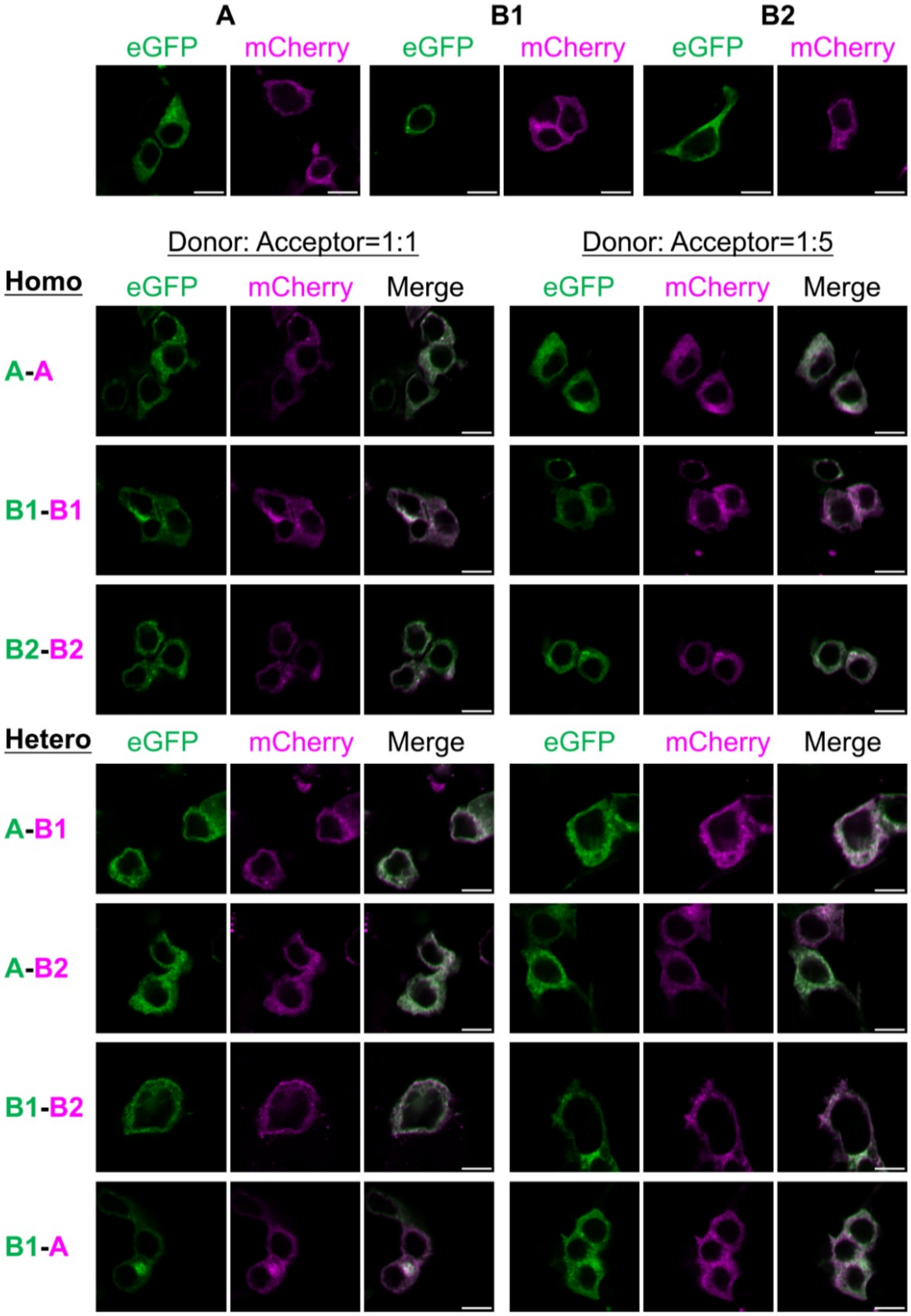

Supplementary Figure 3. Membrane expression of single and double transfected Mc4r.

Mc4r A, B1, B2 expression, by confocal microscopy. Single transfection of eGFP or mCherry fused Mc4r isoforms all show clear cell membrane expression. Double transfection of two Mc4r isoforms with one eGFP tag (donor) and one mCherry tag (acceptor) also exhibit correct cell membrane expression. Scale bar: 10 μm.

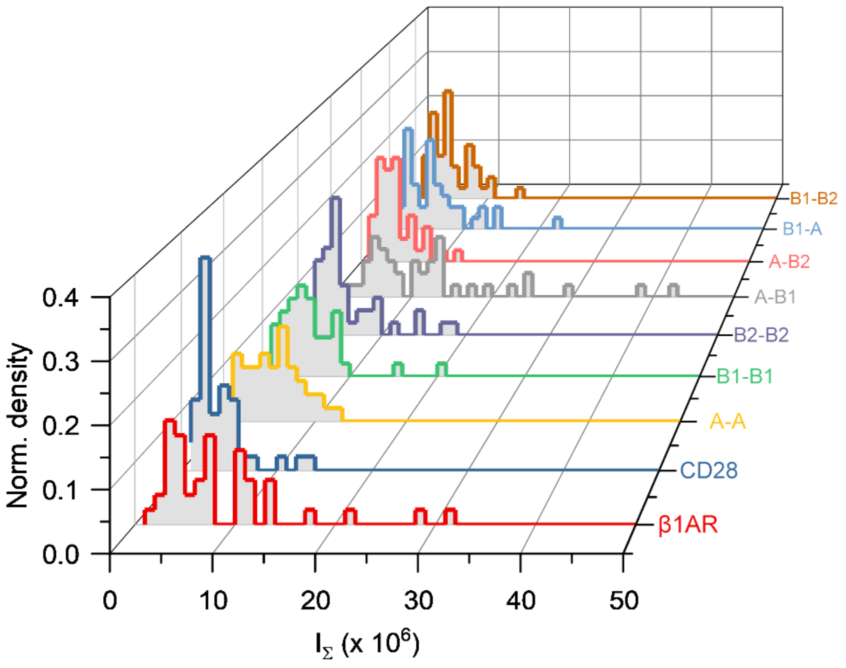

Supplementary Figure 4. Total fluorescent signal intensity of all protomers combinations.

Total fluorescent signal intensity I_{Σ} were calculated by **equation (7)**, from all measured cells. Histogram were plotted with 50 bins for each protomer combination. All I_{Σ} were distributed within one order of magnitude.

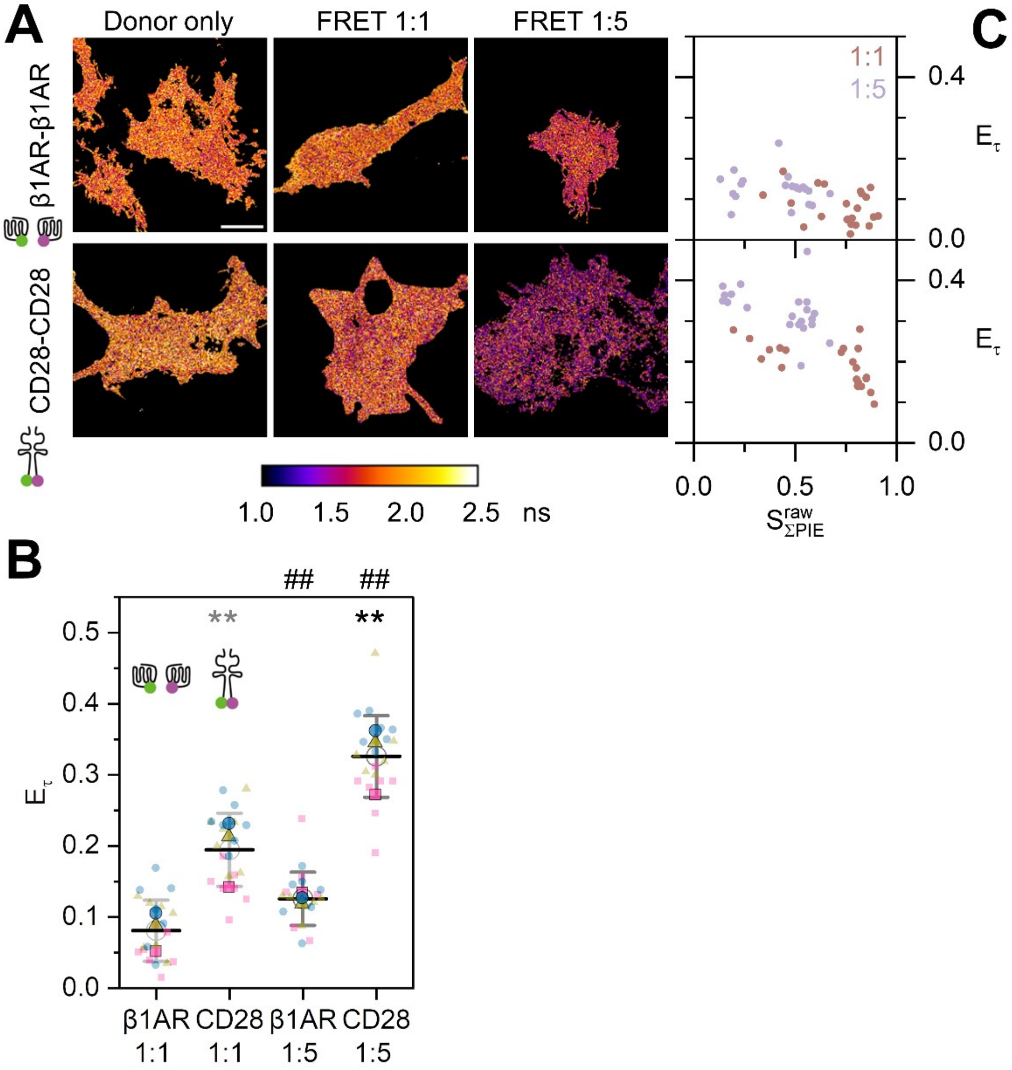

Supplementary Figure 5. Monomer β1AR and dimer CD28 controls.

(A) Lifetime images show species-weighted average lifetime of eGFP. Scale bar: 10 μm.

(B) Superplot of lifetime-based FRET efficiency E_τ. In the superplot, data from three replicates are plotted in red squares, yellow triangles, and blue circles, respectively. The mean of each replicate is indicated with the larger sign of same color pattern. The overall mean (horizontal bar), and standard deviation of the mean (error bars) are indicated. Two tails unpaired *t*-test are used in statistical analysis. Grey *: compared to β1AR 1:1. Black *: compared to β1AR 1:5. Black #: 1:1 *vs.* 1:5 for each pair. Statistics (summary in **Supplementary Table 2**): ns: not significant, */#: $p<0.05$, **/##: $p<0.01$.

(C) 2D scatter plots of lifetime-based FRET efficiency to effective stoichiometry for controls.

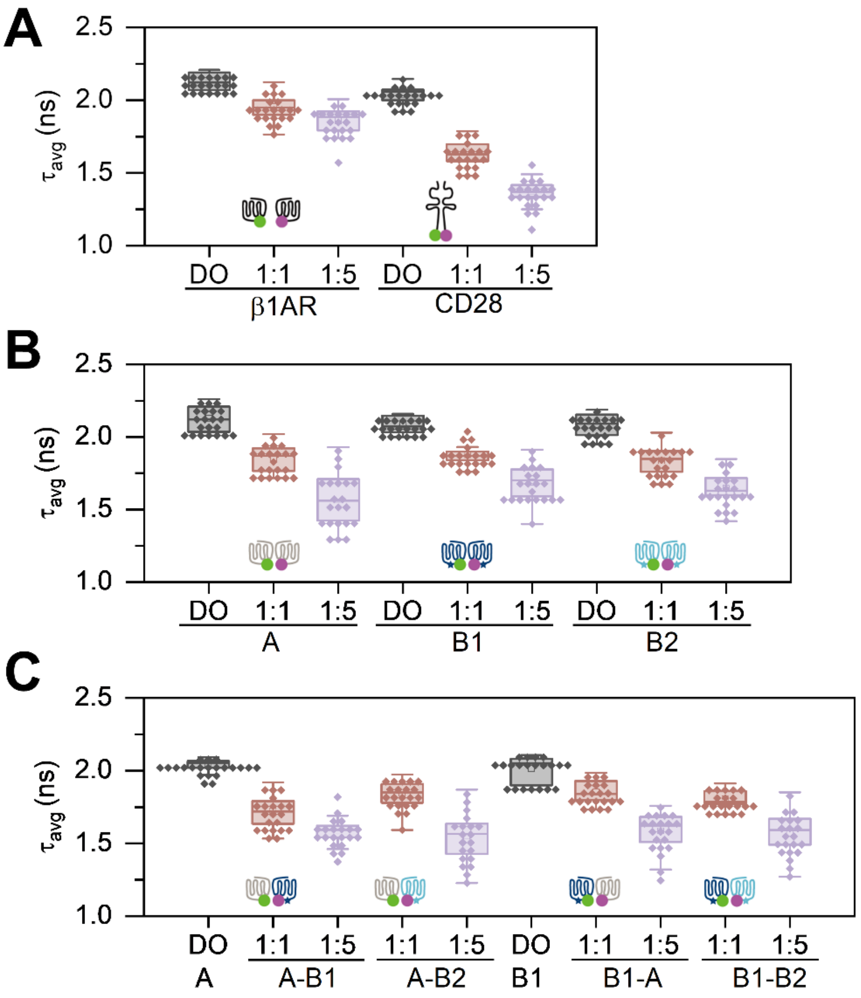

Supplementary Figure 6. Average fluorescence lifetime for two controls and all fish samples.

(A) Controls.

(B) Mc4r homodimers.

(C) Mc4r heterodimers.

In each pair, donor-only samples (black), and samples of donor/acceptor ratio 1:1 (dark orange) and 1:5 (light purple) were displayed. All data are presented as individual points, and the boxes represented median with 25th and 75th percentiles, with whisker at 1.5 interquartile range.

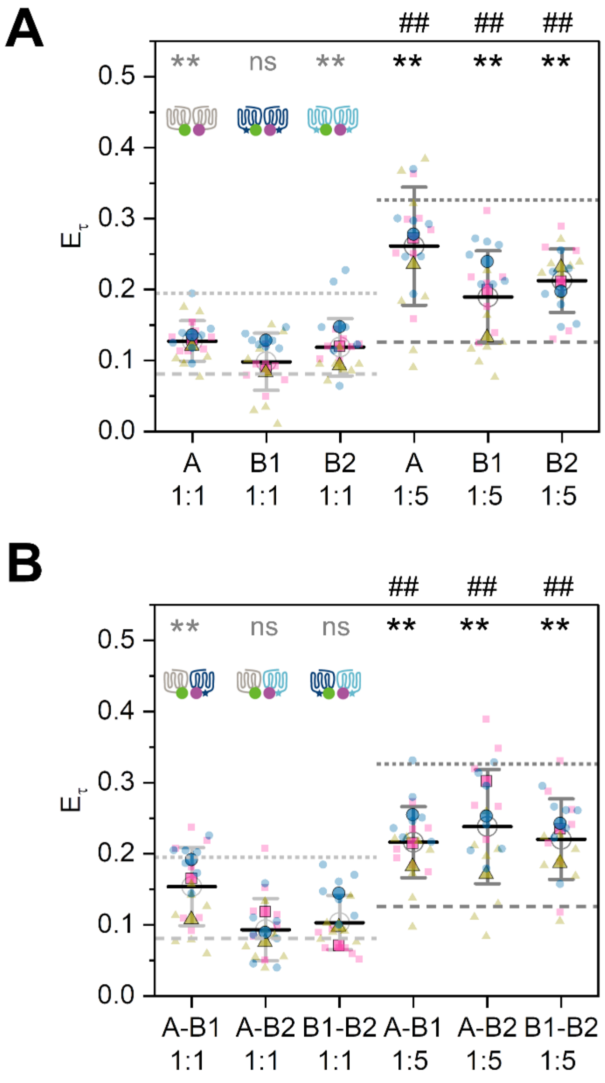

Supplementary Figure 7. Superplots of Mc4r homodimer and heterodimer pairs.

(A) Mc4r homodimers.

(B) Mc4r heterodimers.

Superplot of lifetime-based FRET efficiency E_τ. The superplots were designed same as in **Supplementary Figure 5B**. Dotted and dashed lines represent reference lines from CD28 and β1AR, respectively, at 1:1 (grey) and 1:5 (black). Two tails unpaired *t*-test are used in statistical analysis. Grey *: compared to β1AR 1:1. Black *: compared to β1AR 1:5. Black #: 1:1 *vs.* 1:5 for each pair. Statistics (summary in **Supplementary Table 2**): ns: not significant, */#: p<0.05, **/##: p<0.01.

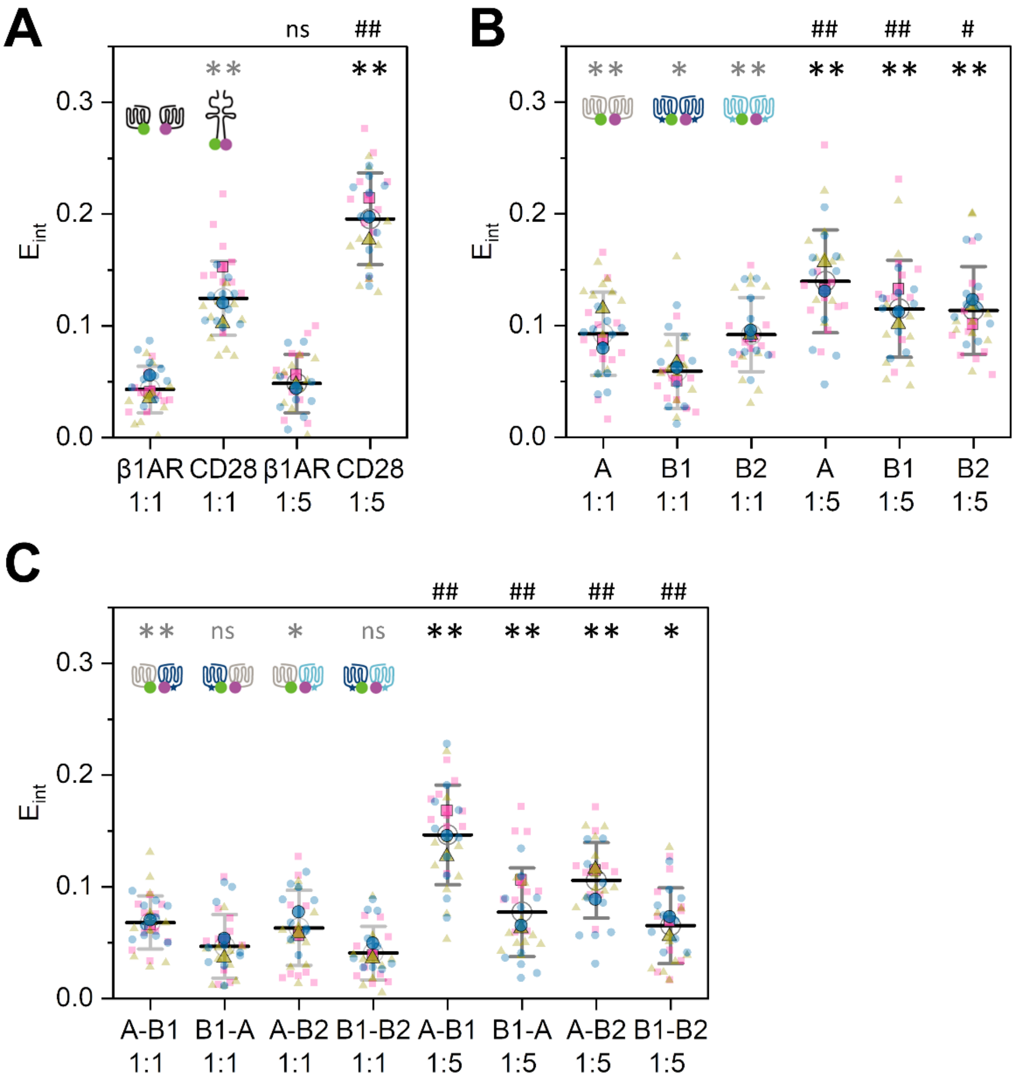

Supplementary Figure 8. FRET-AB measurement of various receptors combinations.

(A) Controls.

(B) Mc4r homodimers.

(C) Mc4r heterodimers.

In each pair, samples with donor/acceptor ratio 1:1 and 1:5 were measured. Superplots were designed same as **Supplementary Figure 5B**. Two tails unpaired *t*-test are used in statistical analysis. Grey *: compared to β1AR 1:1. Black *: compared to β1AR 1:5. Black #: 1:1 *vs.* 1:5 for each pair. Statistics (summary in **Supplementary Table 3**): ns: not significant, */#: p<0.05, **/##: p<0.01.

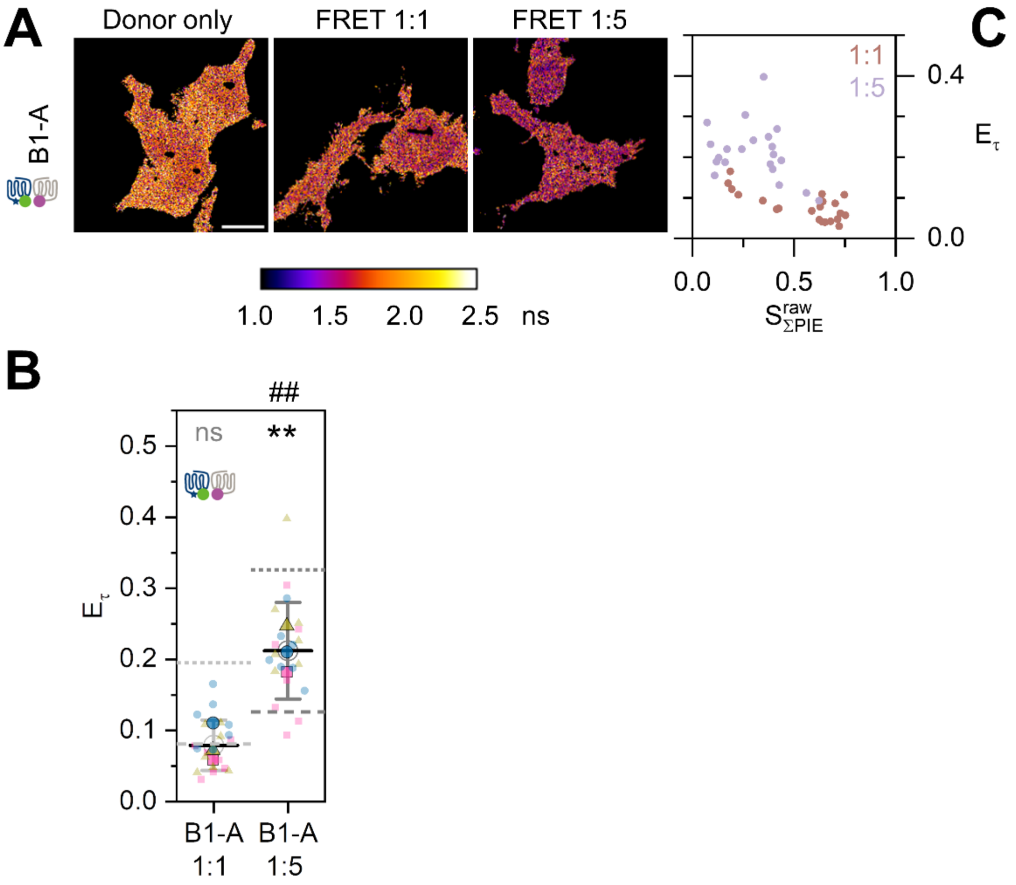

Supplementary Figure 9. Fish Mc4r B1-A heterodimerization.

(A) Lifetime images show species-weighted average lifetime of eGFP. Scale bar: 10 µm.

(B) Superplot of lifetime-based FRET efficiency E_τ. The superplots were designed same as in **Supplementary Figure 5B, Supplementary Figure 7A**. Dotted and dashed lines represent reference lines from CD28 and β1AR, respectively, at 1:1 (grey) and 1:5 (black). Two tails unpaired *t*-test are used in statistical analysis. Grey *: compared to β1AR 1:1. Black *: compared to β1AR 1:5. Black #: 1:1 *vs.* 1:5 for each pair. Statistics (summary in **Supplementary Table 2**): ns: not significant, */#: p<0.05, **/##: p<0.01.

(C) 2D scatter plot of lifetime-based FRET efficiency to effective stoichiometry for B1-A.

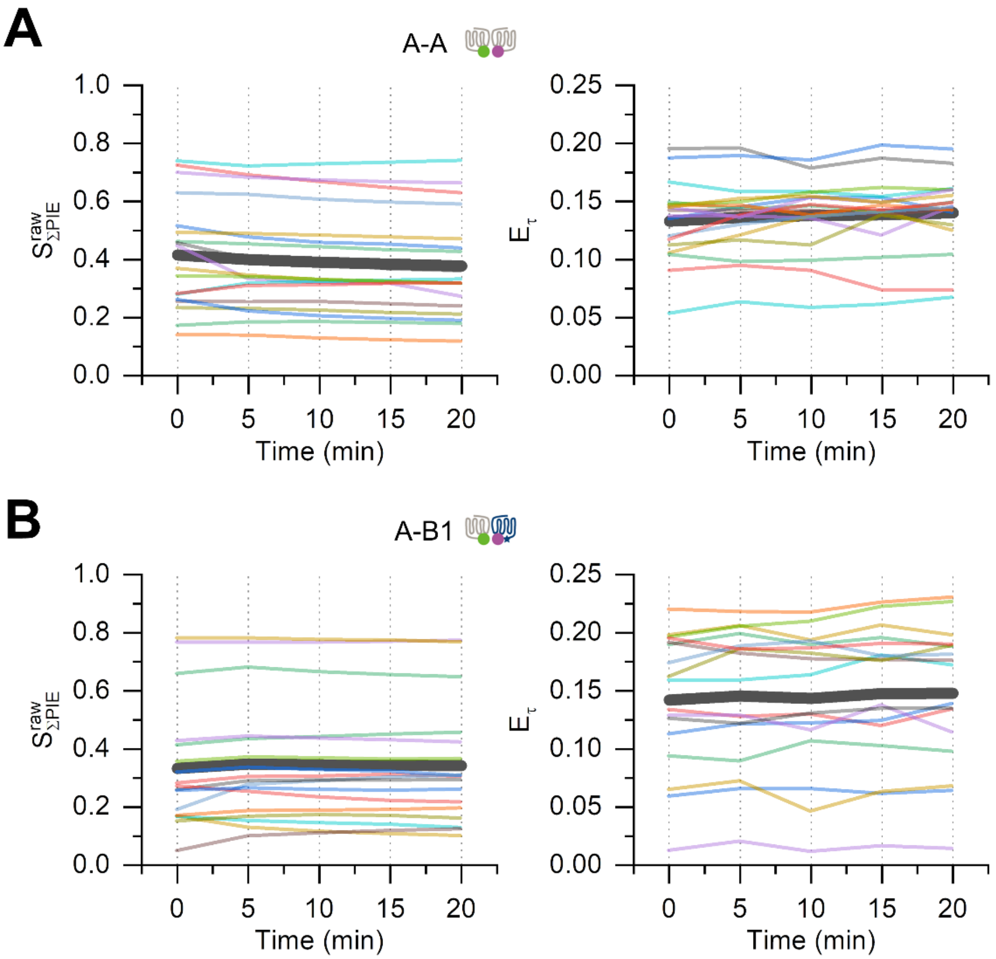

Supplementary Figure 10. Analysis of activated homo- and heterodimers.

(A) Samples A-A with donor/acceptor ratio 1:1.

(B) Samples A-B1 with donor/acceptor ratio 1:1.

Time-course changes were plotted for each cell in transparent thin color lines and the average of all cells are plotted as the thick black lines. Left panels are change of stoichiometry through time and right panels are change of lifetime-based FRET efficiency through time.

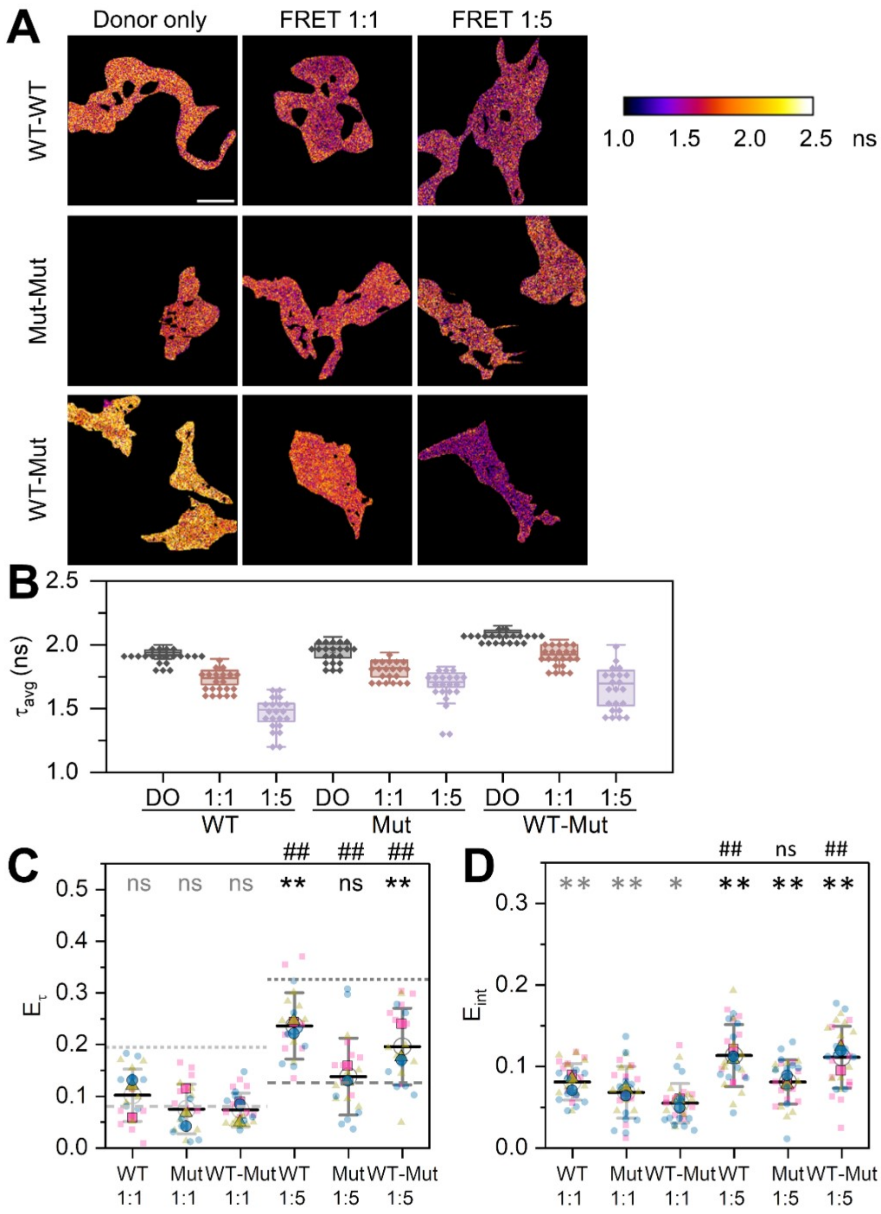

Supplementary Figure 11. Dimerization of human MC4R as a comparison.

(A) Lifetime images show species-weighted average lifetime of eGFP. Scale bar: 10 µm.

(B) Lifetime plots.

(C) FRET-FLIM measurement.

(D) FRET-AB measurement.

Superplots were designed as **Supplementary Figure 7 and Supplementary Figure 8**. Two tails unpaired *t*-test are used in statistical analysis. Grey *: compared to β1AR 1:1. Black *: compared to β1AR 1:5. Black #: 1:1 *vs.* 1:5 for each pair. Statistics (summary in **Supplementary Table 2 and Supplementary Table 3**): ns: not significant, */#: p<0.05, **/##: p<0.01.

1.2 Supplementary Tables

Supplementary Table 1. Primers.

Primers	Sequences	Comments
hMC4R_HindIII_kATG_F	CACC**AAGCTT**GCCGCCATG GTGAACTCCACCCACCGTG	Cloning for human MC4R wild-type/mutant
hMC4R_XbaI_R	TATA**TCTAGA**ATATCTGCT AGACAAGTCAC	Cloning for human MC4R wild-type/mutant
Mc4rA_HindIII_kATG_F	CACC**AAGCTT**GCCGCCATG AACTCCACGGCTCAGCAAG	Cloning for *Xiphophorus* Mc4r A/B1/B2
Mc4rA_XbaI_R	TATA**TCTAGA**CAGAAAGCT AATACACGAGA	Cloning for *Xiphophorus* Mc4r A
Mc4rB1_XbaI_R	TATA**TCTAGA**CAGAAAGCT AATACACCAGA	Cloning for *Xiphophorus* Mc4r B1
Mc4rB2_XbaI_R	TATA**TCTAGA**CAGATGAAC TACCTGGAGCG	Cloning for *Xiphophorus* Mc4r B2
CD28_HindIII_kATG_F	CACC**AAGCTT**GCCGCCATG CTCAGGCTGCTCTTGGCTC	Cloning for CD28
CD28_XbaI_R	TATA**TCTAGA**CCTGCTCCT CTTACTCCTCA	Cloning for CD28
b1AR_HindIII_kATG_F	CACC**AAGCTT**GCCGCCATG GGCGCGGGGGTGCTCGTCC	Cloning for β1AR
b1AR_XbaI_R	TATA**TCTAGA**CACCTTGGA TTCCGAGGCGA	Cloning for β1AR

Note: Red letters indicate restriction sites and blue letter are the Kozak sequence.

Supplementary Table 2. FRET-FLIM statistics summary.

		E_τ	±	N	*t*-test 1:1 *vs.* 1:5		*t*-test each *vs.* β1AR	
β1AR	1:1	0.081	0.043	21	8.60E-04	***		
	1:5	0.126	0.038	21				
CD28	1:1	0.195	0.051	21	1.43E-09	****	1.61E-09	****
	1:5	0.326	0.057	21			2.26E-16	****
A	1:1	0.127	0.029	21	2.01E-08	****	1.88E-04	***
	1:5	0.261	0.083	21			3.49E-08	****
B1	1:1	0.098	0.040	21	2.73E-06	****	1.88E-01	ns
	1:5	0.189	0.065	21			3.79E-04	***
B2	1:1	0.119	0.040	21	1.26E-08	****	5.46E-03	**
	1:5	0.212	0.045	21			3.55E-08	****
A-B1	1:1	0.154	0.055	21	4.08E-04	***	2.31E-05	****
	1:5	0.216	0.050	21			6.00E-08	****
B1-A	1:1	0.079	0.036	21	9.80E-10	****	8.92E-01	ns
	1:5	0.212	0.068	21			8.80E-06	****
A-B2	1:1	0.093	0.044	21	1.08E-08	****	3.61E-01	ns
	1:5	0.238	0.080	20			1.06E-06	****
B1-B2	1:1	0.103	0.038	21	1.21E-09	****	8.44E-02	ns
	1:5	0.221	0.057	21			1.37E-07	****
WT	1:1	0.102	0.051	21	3.74E-09	****	1.52E-01	ns
	1:5	0.236	0.064	21			3.70E-08	****
Mut	1:1	0.075	0.048	20	2.80E-03	**	6.96E-01	ns
	1:5	0.138	0.074	21			5.03E-01	ns
WT-Mut	1:1	0.074	0.033	22	1.02E-08	****	5.41E-01	ns
	1:5	0.196	0.074	22			3.38E-04	***

Note: E_τ represents average lifetime-based FRET efficiency, and ± indicates SD.

ns $p \geq 0.05$, * $p<0.05$, ** $p<0.01$, *** $p<0.001$, **** $p<0.0001$.

Supplementary Table 3. FRET-AB statistics summary.

		E_{int}	±	N	*t*-test 1:1 *vs.* 1:5		*t*-test each *vs.* β1AR	
β1AR	1:1	0.043	0.021	30	3.89E-01	ns		
	1:5	0.048	0.026	30				
CD28	1:1	0.125	0.033	30	6.82E-10	****	1.88E-16	****
	1:5	0.196	0.041	30			1.16E-23	****
A	1:1	0.093	0.037	33	3.48E-05	****	2.24E-08	****
	1:5	0.140	0.046	30			2.67E-13	****
B1	1:1	0.059	0.033	30	6.10E-07	****	2.95E-02	*
	1:5	0.115	0.043	30			1.37E-09	****
B2	1:1	0.092	0.033	30	2.52E-02	*	5.46E-09	****
	1:5	0.113	0.039	30			3.41E-10	****
A-B1	1:1	0.068	0.024	30	8.16E-12	****	6.06E-05	****
	1:5	0.147	0.045	30			6.71E-15	****
B1-A	1:1	0.047	0.028	30	1.09E-03	**	5.69E-01	ns
	1:5	0.077	0.040	30			1.47E-03	**
A-B2	1:1	0.063	0.034	30	8.05E-06	****	7.10E-03	*
	1:5	0.106	0.034	30			7.17E-10	****
B1-B2	1:1	0.041	0.024	30	1.93E-03	**	6.79E-01	ns
	1:5	0.065	0.034	30			3.49E-02	*
WT	1:1	0.081	0.022	30	1.69E-04	***	5.49E-09	****
	1:5	0.113	0.038	30			1.87E-10	****
Mut	1:1	0.068	0.032	30	1.01E-01	ns	5.70E-04	***
	1:5	0.081	0.027	30			1.40E-05	****
WT-Mut	1:1	0.055	0.024	33	1.32E-09	****	4.00E-02	*
	1:5	0.111	0.038	31			3.37E-10	****

Note: E_{int} represents intensity-based FRET efficiency, and ± indicates SD.

ns $p \geq 0.05$, * $p < 0.05$, ** $p < 0.01$, *** $p < 0.001$, **** $p < 0.0001$.

2 Supplementary Data

Supplementary Script 1. Export Symphotime-based ROIs for grouped/ensemble analysis.

```
###################################
# Katherina Hemmen ~ Core Unit Fluorescence Imaging ~ RVZ
# katherina.hemmen@uni-wuerzburg.de
###################################

# Converting Symphotime ROIs export for grouped / ensemble analysis

# Input:
# (1) ASCII export of green channel data, where the ROI has been marked in Symphotime
# Data is in txt format, where the top-half of the file corresponds to the green
# photon count and the bottom-half corresponds to the tau(FastFLIM) calculated by Symphotime
# (2) Tif-images exported from Symphotime for the following channels and time windows:
# (i) green prompt, (ii) red prompt and (iii) red delay

# Output:
# (1) Intensity images of the three channels & time windows
# (i) green prompt, (ii) red prompt and (iii) red delay
# where intensity values outside the marked ROI have been set to 0
# (2) Table of summarizing values, with first column being the filename:
# (i) sum of number of green photons in prompt time range (gp)
# (ii) sum of number of red photons in prompt time range (rp)
# (iii) sum of number of red photons in delay time range (rd)
# (iv) green to red intensity ratio in prompt time window
# (v) effective stoichiometry
# (vi) apparent FRET efficiency (proximity ratio)

# 1. Importing of all required modules
from skimage import io
import glob
import os
import numpy as np

# 2. Data to be processed
path = 'C:/Users/Downloads/DATA/*.ptu'
# All files within this folder in PTU format will be used to generate a list of filenames.
save_file_as = 'C:/Users/Downloads/Results.txt'
# The relevant parameter such as sum of photons are saved into a txt document.

# Initialize list of parameters to be saved at the end
list_filenames = list()  # filenames
list_nr_green_ph = list()  # number of green photons in prompt time range (gp)
list_nr_red_p_ph = list()  # number of red photons in prompt time range (rp)
list_nr_red_d_ph = list()  # number of red photons in delay time range (rd)
list_SgSr = list()  # green to red intensity ratio in prompt time window
list_effSpie = list()  # effective stoichiometry
list_appE = list()  # apparent FRET efficiency (proximity ratio)

# Loop over all files in folder
for file in glob.glob(path):
filename = os.path.abspath(file).split(".")[0]  # Get the filenames

print('Processing....' + filename)

# 3. Read and convert Symphotime ASCII file into binary ROI
ROI = filename + '.txt'
ROI_int = np.genfromtxt(ROI, skip_header=3, skip_footer=259)  # First half of txt file
ROI_mean_tau = np.genfromtxt(ROI, skip_header=262)  #  Second half of txt file
io.imsave(filename + '_green int.tif', ROI_int, check_contrast=False)
io.imsave(filename + '_tauFF.tif', ROI_mean_tau, check_contrast=False)

# ROI_int and ROI_mean_tau have values identical to number of green photons
# / mean lifetime inside the ROI and values = 0 outside the ROI
# To generate the binary ROI (an image filled with 0 outside ROI and 1 inside),
# ROI_int is used and all values > 0 are set to 1
binary_ROI = np.where(ROI_int > 0, 1, 0)
io.imsave(filename + '_ROI.tif', binary_ROI, check_contrast=False)

# 4. Use ROI to generate masks on the intensity images
```

```
# Read the exported tif-files for channels/time windows
# All images have two channels: channel 0 = intensity, channel 1 = tau(FastFLIM)
int_gp = io.imread(filename + '_gp.tif')[0]
int_rp = io.imread(filename + '_rp.tif')[0]
int_rd = io.imread(filename + '_rd.tif')[0]
# All values where the binary ROI = 1 are kept, other pixel values are set 0
masked_intensity_gp = np.where(binary_ROI ==1, int_gp, 0)
masked_intensity_rp = np.where(binary_ROI ==1, int_rp, 0)
masked_intensity_rd = np.where(binary_ROI ==1, int_rd, 0)

# 5. Calculate and save sum of photons
sum_green_ph = np.sum(masked_intensity_gp)
sum_red_prompt_ph = np.sum(masked_intensity_rp)
sum_red_delay_ph = np.sum(masked_intensity_rd)

# 6. Calculate FRET-related parameters
# Note: apparent FRET efficiency and stoichiometry are calculated as no corrections are applied
SgSr = sum_green_ph / sum_red_prompt_ph
effSpie = (sum_green_ph + sum_red_prompt_ph) / (sum_green_ph + sum_red_prompt_ph
+ sum_red_delay_ph)
appE = sum_red_prompt_ph / (sum_red_prompt_ph + sum_green_ph)

# Append all values to a growing list
list_filenames.append(str(file))  # filenames
list_nr_green_ph.append(sum_green_ph)  # number of green photons in prompt time range
list_nr_red_p_ph.append(sum_red_prompt_ph)  # number of red photons in prompt time range
list_nr_red_d_ph.append(sum_red_delay_ph)  # number of red photons in delay time range
list_SgSr.append(SgSr)  # green to red intensity ratio in prompt time window
list_effSpie.append(effSpie)  # effective stoichiometry
list_appE.append(appE)  # apparent FRET efficiency (proximity ratio)

# Column header of saved txt file
header = 'Filename\tNr Green ph\tNr Red ph (prompt)\tNr Red ph (delay)\tSgSr\teffSpie\tappE'

# Save results as txt file
np.savetxt(
save_file_as,
np.vstack(
[
list_filenames,
list_nr_green_ph,
list_nr_red_p_ph,
list_nr_red_d_ph,
list_SgSr,
list_effSpie,
list_appE
]
).T,
delimiter='\t', fmt="%s", header=header
)
```

Supplementary Script 2. Export tables for pixel-wise analysis.

```
###################################
# Katherina Hemmen ~ Core Unit Fluorescence Imaging ~ RVZ
# katherina.hemmen@uni-wuerzburg.de
###################################

# Export for pixel-wise analysis and heterogeneity analysis

# Input: ROI-masked intensity images (green prompt, red prompt, red delay)
# and mean tau green

# Output: Table of 256 x 256 = 65535 lines and the following columns:
# Y pixel - X pixel- Mean Tau(green) - Number of photons(green) - Number of photons(red prompt)
# - Number of photons(red delay) - eff Stoichiometry(PIE) - SgSr - app FRET efficiency

# 1. Importing of all required functions
from skimage import io, img_as_ubyte
import glob
import os
import numpy as np

# 2. Data to be processed
path = 'C:/Users/Downloads/DATA/*.ptu'
# All files within this folder in PTU format will be used to generate a list of filenames.

# Loop over all files in folder
for file in glob.glob(path):
    filename = os.path.abspath(file).split(".")[0]  # Get the filenames
    output_filename = filename + '.er4'  # How to save the results

    print('Processing....' + filename)

    # Donor mean fluorescence lifetime - based on symphotime export
    mean_tau_green_img = filename + '_tauFF.tif'
    mean_tau_green = io.imread(mean_tau_green_img)

    # Intensity images - split by color channel and time windows (prompt & delay)
    green_intensity_img = filename + '_gp.tif'
    green_intensity = io.imread(green_intensity_img)[0]

    red_prompt_intensity_img = filename + '_rp.tif'
    red_prompt_intensity = io.imread(red_prompt_intensity_img)[0]

    red_delay_intensity_img = filename + '_rd.tif'
    red_delay_intensity = io.imread(red_delay_intensity_img)[0]

    # 2. Loading the generated cell mask
    cell_ROI = filename + '_ROI.tif'
    mask = img_as_ubyte(io.imread(cell_ROI))

    # 3. Select the pixels of interest
    tau_green = mean_tau_green * mask
    nD = green_intensity * mask
    nA_FRET = red_prompt_intensity * mask
    nA_direct = red_delay_intensity * mask

    # 4. Calculate the derived parameter
    #----------------------------------------------
    # 4.1 effective Stoichiometry-PIE
    # ^^^^^^^^^^^^^^^^^^^^^^^^^^^^^^^^
    # Note: effective Spie as we omit all correction factors

    Spie = (nA_FRET + nD) / (nA_FRET + nD + nA_direct)

    # 4.2 Green-Red intensity ratio, Sg/Sr
    # ^^^^^^^^^^^^^^^^^^^^^^^^^^^^^^^^^^^^
    # ~> related also to FRET efficiency, but uncorrected intensities are used

    SgSr = nD / nA_FRET

    # 4.3 apparent FRET-efficiency (proximity ratio) based on intensities
```

```
    # ^^^^^^^^^^^^^^^^^^^^^^^^^^^^^^^^^^^^^^^^^^^^^^^^
    # For correct FRET efficiency, a correction factor is required: gamma
    # Gamma defines the ratio of the detector sensitivities
    # E = nA(FRET) /(nA(FRET) + gamma * nD) <=> Eapp = nA(FRET) /(nA(FRET) + nD)

    Eapp = nA_FRET / (nA_FRET + nD)

    # 5. Reshape the parameter matrices
    # All parameters are reshaped from a 256 x 256 array -> 1 x 65'536 array
    lines, pixel = mean_tau_green.shape
    tau_green_1D = tau_green.reshape((lines * pixel))
    nD_1D = nD.reshape((lines * pixel))
    nA_FRET_1D = nA_FRET.reshape((lines * pixel))
    nA_direct_1D = nA_direct.reshape((lines * pixel))
    Spie_1D = Spie.reshape((lines * pixel))
    SgSr_1D = SgSr.reshape((lines * pixel))
    Eapp_1D = Eapp.reshape((lines * pixel))

    # 6. Generate a list of pixel coordinates
    pixel_id = np.indices((lines, pixel))
    pixel_id_1D = pixel_id.reshape((2, lines*pixel))
    Y_pixel = np.array(pixel_id_1D[0, :])
    X_pixel = pixel_id_1D[1, :]

    # 7. Export results as text files
    # These files can be read by any text editor or e.g. Margarita from the Seidel-Software
Package
    header = "Y pixel\tX pixel\tMean Tau(green)\tNumber of photons(green)\tNumber of photons(red
prompt)\tNumber of photons(red delay)\teff Stoichiometry(PIE)\tSgSr\tapp FRET efficiency"

    # Change the saving directory!
    np.savetxt(
        output_filename,
        np.vstack([Y_pixel, X_pixel, tau_green_1D, nD_1D, nA_FRET_1D, nA_direct_1D, Spie_1D,
SgSr_1D, Eapp_1D]).T,
        delimiter='\t',
        header=header
        )
```

3 Supplementary Information

Xiphophorus multilineatus Mc4r A

>Xmulti_Mc4r_A_nt

ATGAACTCCACGGCTCAGCAAGGCTTGATCCCATGCTACCTGAACGGGAGCCTGTGCCCGGGAACTC
TGCCAGAGAAAGACGTTTCCGGAGAGGAGAAGGATTCCTCTGCTGGCTGCTCTGAGCAGCTGCTGAT
TTCCACCGAGGTCTTCCTCACTTTGGGCATCATCAGCCTGCTGGAGAACATCCTAGTGGTCGCCGCC
ATCATCAAGAACCAGAACCTCCATTCCCCGATGTACTTCTTCATCTGCAGCCTGGCGGTTGCTGACA
TGCTGGTCAGCGTCTCCAACGCCTCGGAGACGATCGTCATAGCGCTGATCAACGGCGGCAGCCTCAC
CATCCCCGTCACGTTCATCAAAAGCATGGACAATGTGTTCGACTCCATGATCTGCAGCTCTCTGCTC
GCCTCCATCTGCAGCTTGCTCGCCATCGCCATCGACCGCTACATCACCATCTTCTACGCCCTGCGGT
ACCACAACATCGTCACCATCCGGCGGGCGTTGCTGGTCATCGCCGGCATCTGGACGTGCTGCACCGT
CTCTGGCATCCTGTTCATCATCTACTCGGAGAGCACCATGGTGCTCATCTGCCTCATCACCATGTTC
TTCACCATGCTGGTCCTCATGGCGTCGCTCTACGTTCACATGTTCCTGCTGGCGCGCCAGCACATGA
AGCGCATCGCCGCCCTACCGGGCAACGCACCCATCCAGCAGCGCGCCAACATGAAGGGCGCCATCAC
CCTCACCATCCTGCTGGGGGTGTTTGTGGTGTGCTGGGCGCCCTTCTTTCTGCACCTCATCCTGATG
ATCACCTGCCCCAGGAACCCCTACTGCACCTGCTTCATGTCGCACTTCAACATGTACCTCATCCTCA
TCATGTGCAACTCCATCATTGACCCCATCATCTACGCTTTCCGCAGCCAGGAGATGAGGAAGACCTT
TAAGGAGATCTTCTGCTGCTCACAGGCTCTCTCGTGTATTAGCTTTCTGTGA

>Xmulti_Mc4r_A_aa

MNSTAQQGLIPCYLNGSLCPGTLPEKDVSGEEKDSSAGCSEQLLISTEVFLTLGIISLLENILVVAA
IIKNQNLHSPMYFFICSLAVADMLVSVSNASETIVIALINGGSLTIPVTFIKSMDNVFDSMICSSLL
ASICSLLAIAIDRYITIFYALRYHNIVTIRRALLVIAGIWTCCTVSGILFIIYSESTMVLICLITMF
FTMLVLMASLYVHMFLLARQHMKRIAALPGNAPIQQRANMKGAITLTILLGVFVVCWAPFFLHLILM
ITCPRNPYCTCFMSHFNMYLILIMCNSIIDPIIYAFRSQEMRKTFKEIFCCSQALSCISFL*

Xiphophorus multilineatus Mc4r B1

>Xmulti_Mc4r_B1_nt

ATGAACTCCACGGCTCAGCAAGGCTTGATCCCATGCTACCTGAACGGGAGCCTGTGCCCGGGAACTC
TGCCAGAGAAAGACGTTTCCGGAGAGGAGAAGGATTCCTCTGCTGGCTGCTCTGAGCAGCTGCTGAT
TTCCACCGAGGTCTTCCTCACTTTGGGCATCATCAGCCTGCTGGAGAACATCCTAGTGGTCGCCGCC
ATCATCAAGAACAAGAACCTCCATTCCCCGAAGTACTTCTTCATCTGCAGCCTGGCGGTCGCTGACA
TGCTGGTCAGCGTCTCCAACGCCTCGGAGACAATCGTCATAGCGCTGTTCAACGGCGGCAGCCTCAC
CATCCCTGTCACGTTCATCAAAAGCATGGACAATGTGTTCAACTCCATGATCTGCAGCTCTCTGCTC
GCCTCCATCTGCAGCTTGCTCGCCATCGCCATCGACCGCTACATCACCATCTTCTACGCCCTGCGGT
ACCACAACATCGTCACCATCCGGCGGGCGTTGCTGGTCATCGGCAGCATCTGGACGTGCTGCACCGT
CTCTGGCATCCTGTTCATCATCTACTCGGAGAGCACAGTGGTGCTCATCTGCCTCATCACCATGTTC
TTCACCGTGCTGGTCCTCATGGCGTCGCTCTACGTTCACATGTTCCTGCTGGCGCGCCAGCACATGA
AGCGCATCGGCGCCCTACCGGGCAACGCGGCCATCCAGCAGCGCGCCAACATGAAGGGCGCCATCAC
CCTCACCATCCTGCTGGGGGTGTTTGTGTTTTGCTGGGCGCCCTTCTTTCTGCACCTCATCCTGATG
ATCACCTGCCCCAGGAACCCCTACTGCACCTGCTTCATGTCGCACTTCAACATGTACCTCATCCTCA

TCATGTGCAACTCCGTCATCGACCCCATCATCTACGCTTTCCGCAGCCAGGAGATGAGGAAGACCTT
TAAGAAGATCTTCTCACAGGCTCTCTGGTGTATTAGCTTTCTGTGA

>Xmulti_Mc4r_B1_aa

MNSTAQQGLIPCYLNGSLCPGTLPEKDVSGEEKDSSAGCSEQLLISTEVFLTLGIISLLENILVVAA
IIKNKNLHSPKYFFICSLAVADMLVSVSNASETIVIALFNGGSLTIPVTFIKSMDNVFNSMICSSLL
ASICSLLAIAIDRYITIFYALRYHNIVTIRRALLVIGSIWTCCTVSGILFIIYSESTVVLICLITMF
FTVLVLMASLYVHMFLLARQHMKRIGALPGNAAIQQRANMKGAITLTILLGVFVFCWAPFFLHLILM
ITCPRNPYCTCFMSHFNMYLILIMCNSVIDPIIYAFRSQEMRKTFKKIFSQALWCISFL*

Xiphophorus multilineatus Mc4r B2

>Xmulti_Mc4r_B2_nt

ATGAACTCCACGGCTCAGCAAGGCTTGATCCCATGCTACCTGAACGGGAGCCTGTGCCCGGGAACTC
TGCCAGAGAAAGACGTTTCCGGAGAGGAGAAGGATTCCTCTGCTGGCTGCTCTGAGCAGCTGCTGAT
TTCCACCGAGGTCTTCCTCACTTTGGGCATCATCAGTCTGCTGGAGAACATCCTAGTGGTCGCCGCC
ATCATCAAGAACAAGAACCTCCATTCCCCGAAGTACTTCTTCATCTGCAGCCTGGCGGTCGCTGACA
TGCTGGTCAGCGTCTCCAACGCCTCGGAGACAATCGTCATAGCGCTGTTCAACGGCGGCAGCCTCAC
CATCCCTGTCACGTTCATCAAAAGCATGGACAATGTGTTCAACTCCATGATCTGCAGCTCTCTGCTC
GCCTCCATCTGCAGCTTGCTCGCCATCGCCATCGACCGCTACATCACCATCTTCTACGCCCTGCGGT
ACCACGACATCGTCACCATCCGGCGGGCGTTGCTGGTCATCGGCAGCATCTGGACGTGCTGCACCGT
CTCTGGCATCCTGTTCATCATCTACTCGGAGAGCACAGTGGTGCTCATCTGCCTCATCACCATGTCC
TTCACCGTGCTGGTCCTCATGGCGTCGCTCTACGTTCACATGTTCCTGCTGGCGCGCCAGCACATGA
AGCGCATCGGCGCCCTACCGGGCAACGCGGCCATCCAGCAGCGCGCCAACATGAAGGGCGCCATCAC
CCTCACCATCCTGCTGGGGGTGTTTGTGTTTTGCTGGGCGCCCTTCTTTCTGCACCTCATCCTGATG
ATCACCTGCCCCAGGAACCCCTACTGCACCTGCTTCATGTCGCACTTCAACATGTACCTCATCCTCA
TCATGTGCAACTCCGTCATCGACCCCATCATCTACGCTTTCCGCAGCCAGGAGATGAGGAAGACCTT
TAAGAAGATCTTCTCACAGGCTCGTGTATTAGCTTTCTGTGAGACGCTCCAGGTAGTTCATCTGTAA

>Xmulti_Mc4r_B2_aa

MNSTAQQGLIPCYLNGSLCPGTLPEKDVSGEEKDSSAGCSEQLLISTEVFLTLGIISLLENILVVAA
IIKNKNLHSPKYFFICSLAVADMLVSVSNASETIVIALFNGGSLTIPVTFIKSMDNVFNSMICSSLL
ASICSLLAIAIDRYITIFYALRYHDIVTIRRALLVIGSIWTCCTVSGILFIIYSESTVVLICLITMS
FTVLVLMASLYVHMFLLARQHMKRIGALPGNAAIQQRANMKGAITLTILLGVFVFCWAPFFLHLILM
ITCPRNPYCTCFMSHFNMYLILIMCNSVIDPIIYAFRSQEMRKTFKKIFSQARVLAFCETLQVVHL*
